# Neurotransmitter‐Mimicking Nanovesicles Facilitate Postoperative Glioblastoma Stem Cell‐Specific Treatment for Preventing Tumor Recurrence

**DOI:** 10.1002/advs.202409713

**Published:** 2024-12-25

**Authors:** Fuming Liang, Qing You, Bin Yu, Chen Wang, Yanlian Yang, Ling Zhu, Zhaohui He

**Affiliations:** ^1^ Department of Neurosurgery The First Affiliated Hospital of Chongqing Medical University 1 Friendship Road Chongqing 400016 P. R. China; ^2^ CAS Key Laboratory of Standardization and Measurement for Nanotechnology CAS Key Laboratory for Biomedical Effects of Nanomaterials and Nanosafety CAS Center for Excellence in Nanoscience National Center for Nanoscience and Technology Beijing 100190 P. R. China; ^3^ University of Chinese Academy of Sciences Beijing 100049 P. R. China; ^4^ Department of Diagnostic Radiology Yong Loo Lin School of Medicine National University of Singapore Singapore 119074 Singapore; ^5^ Department of Radiology The First Affiliated Hospital of Chongqing Medical University 1 Friendship Road Chongqing 400016 P. R. China

**Keywords:** D2DR, glioblastoma recurrences, glioblastoma stem cell inhibitions, neurotransmitter mimickings

## Abstract

Survival quality of glioblastoma (GBM) patients remains undesirable despite the aggressive multimodal treatment methods implemented, which are strongly associated with tumor recurrence after surgical resection. Self‐renewal and strong tumourigenic capacity of glioblastoma stem cells (GSCs) at the narrow margin of the incision are essential factors driving tumor secondary strikes. Currently, the challenges in treating postoperative residual GSCs are mainly due to the lack of materials for incision and GSCs targeting. In this study, a neurotransmitter‐mimicking nanovesicle (PMVS‐P) based on platelet membrane‐derived vesicle (PMV) with anti‐GSC drug salinomycin (SAL)‐loading and polydopamine (PDA)‐surface is synthesized. PMVS‐P exhibits surgical incision targeting ability and specifically identified GSCs with highly expressed D2 dopamine receptor (D2DR), a central nervous system neurotransmitter receptor, thus suppressing GBM recurrence. This neurotransmitter‐mimicking nanovesicle primed GSC‐specific tumoricidal treatment with broadened applications for preventing tumor recurrence.

## Graphical abstract

Neurotransmitter‐mimicking nanovesicles are developed for cancer stem cell‐specific tumoricidal treatment to prevent glioblastoma recurrence. These nanovesicles exhibit surgical incision aggregation and target postoperative residual cancer stem cells through D2 dopamine receptor (D2DR) identification.

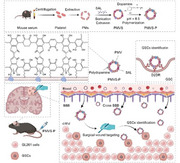

## 1 Introduction

Glioblastoma (GBM) is the most highly malignant brain tumor with a short survival period and a high recurrence rate.^[^ ^1^ ^]^ The current standard treatment of GBM is primary surgical resection along with adjuvant radiotherapy. However, a large proportion of GBM recurs in the narrow margins of the resected cavity, directly leading to a poor prognosis and unsatisfied therapeutic outcomes.^[^ ^2^ ^]^ Glioblastoma stem cells (GSCs) are tumor‐initiating cells with strong tumorigenicity and self‐renewal capacity which are present in residual tumor tissues after surgery and drive GBM recurrence.^[^ ^3^ ^]^ Thus, the eradication of postoperative residual GSCs is a key factor in inhibiting GBM recurrence. Studies have shown that GSCs exist at the margins of surgical incisions and mediate the recurrence of GBM from surgical incisions.^[^ ^3^ ^]^ Therefore, incision targeting is an effective strategy for nanomaterials against GBM recurrence. Platelets are a class of incision‐responsive blood cells due to the abundance of membrane proteins such as glycoprotein Ib (GPIb), glycoprotein Ia‐IIa (GPIa‐IIa), and integrins, which bind to incision‐exposed collagen IV, facilitating hemostatic and trophic effects.^[^ ^4^ ^]^ Platelet membrane vesicles (PMVs) with a high abundance of platelet membrane proteins can also exhibit incision‐targeting capabilities. Besides, they also serve as drug‐loading and delivery carriers due to their hydrophobic phospholipid bilayer and hydrophilic cavity.^[^ ^5^ ^]^ The high biocompatibility and low immunogenicity of biologically sourced PMVs also protect the nanovesicles from being cleared by the immune system,^[^ ^6^ ^]^ thus reducing immune rejection of PMVs at the incision site. Therefore, PMV is a promising nanomaterial for targeting residual tumor tissues at the GBM surgical incision site.

In addition to surgical incision targeting, promoting drug identification of GSCs is also a critical factor in inhibiting GBM recurrence. The physiologic D2 dopamine receptor (D2DR) is primarily expressed in the central nervous system and binds to dopamine neurotransmitters to produce neuroexcitation.^[^ ^7^ ^]^ However, studies have found that the high expression of D2DR promotes poor prognosis in GBM due to the maintenance of the dedifferentiated phenotype of GSCs, indicating that D2DR is a target for cancer stem cell therapy.^[^ ^8^ ^]^ Therefore, neurotransmitter dopamine modification of the nanomaterials improves GSC indentification.^[^ ^9^ ^]^ Recently, researchers have found that dopamine‐functionalized nanomaterials can bind to D2DR due to the identification of catechol and amine groups of synthesized polydopamine (PDA), mediating the endocytosis of D2DR‐enriched cells.^[^ ^10^ ^]^ Thus, dopamine‐functionalized nanovesicles may have potential in GSC targeting by D2DR‐mediated identification.

**Scheme 1:**
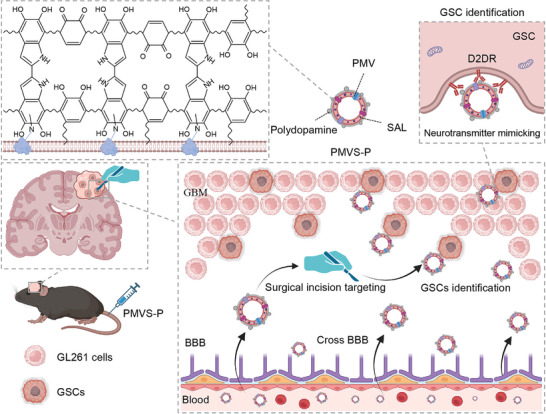
Schematic illustration of the neurotransmitter‐mimicking nanovesicles for GSC inhibition and GBM recurrence prevention. GSCs at residual tumor tissue mediate postoperative recurrence of GBM. PMVS‐P targets surgical incision tissue after BBB crossing and promotes the elimination of GSCs by recognizing highly expressed D2DR, thereby inhibiting GBM recurrence after surgery.

In this work, we synthesized neurotransmitter‐mimicking nanovesicle PMVS‐P by loading anti‐GSCs drug salinomycin (SAL) into the phospholipid bilayer of PMV and arming PDA on its surface via in situ polymerization. PMVs promoted drug aggregation in residual tumor tissue at the GBM surgical incision site. PDA shell targeted GSCs via D2DR‐mediated binding and produced GSCs cytotoxicity in the presence of SAL, thus inhibiting GBM recurrence (**Scheme** **1**). We provide a strategy to effectively target GSCs in postoperative residual lesions, shedding light on a new therapeutic idea for inhibiting GBM recurrence. Our study elucidates the mechanism by which PDA targets tumor stem cells. Since PDA is often used as a direct carrier for drug delivery or as a coating for nanomaterials, our findings also provide a new biological function for widely used PDA‐based nanomaterials for cancer stem cell therapy.

## 2 Results

### 2.1 GSCs are Present in Postoperative Residual Tumor Tissue and Mediate GBM Recurrence

**Figure 1:**
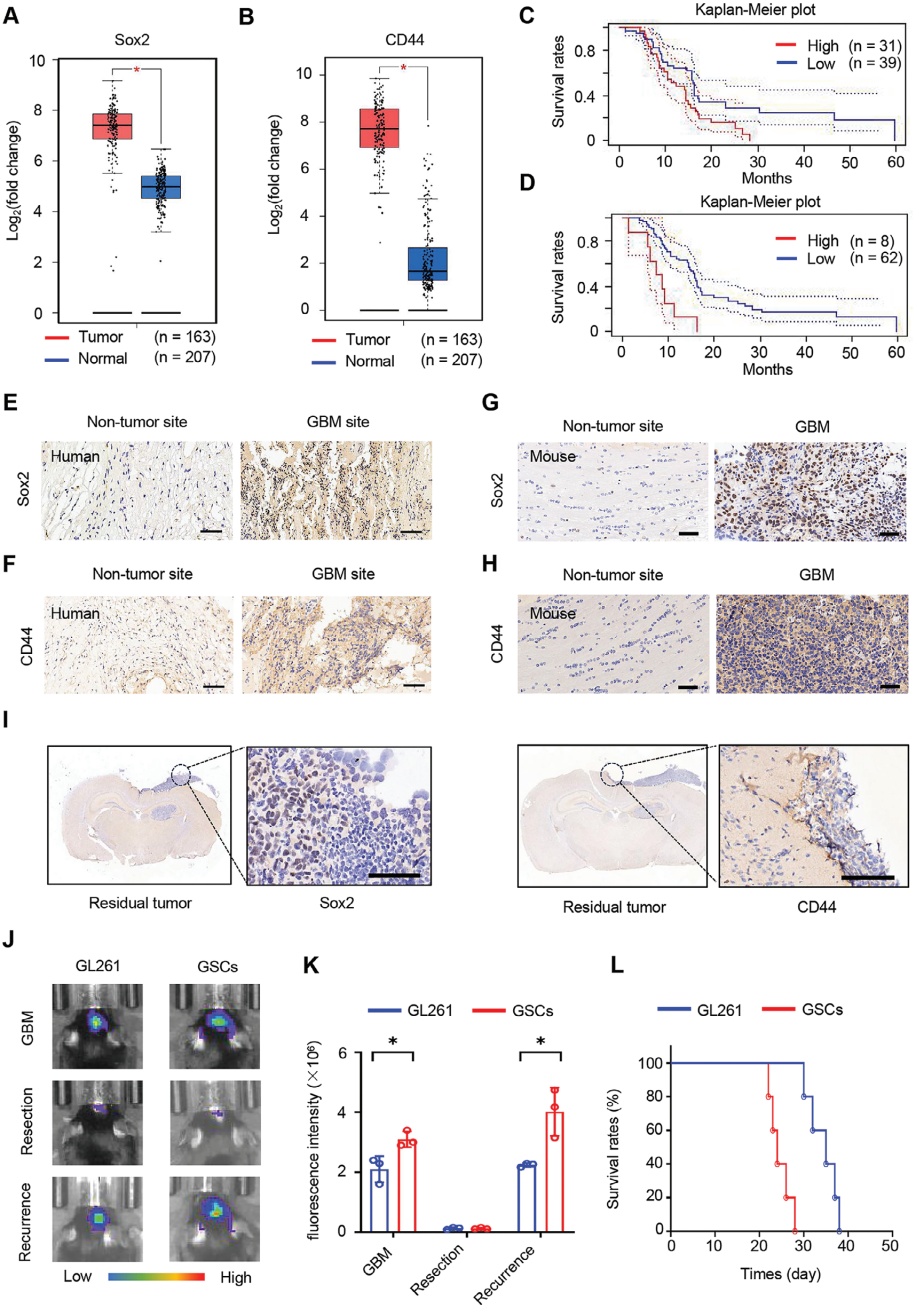
GSCs are present in postoperative residual GBM tissue and promote GBM recurrence. (A‐B) The gene expression of A) Sox2 and B) CD44 in human GBM and normal brain tissues. Statistical significance was analyzed using the one‐way analysis of variance (ANOVA) with Tukey's post hoc test. *p < 0.001. Sox2 and CD44 gene expression platforms in patients with GBM and healthy people were retrieved from The Cancer Genome Atlas. In each analysis, 163 GBM patients and 207 normal controls were randomly selected from the same large sample, so there may be scattered points with same distribution between different analysis of the same marker. Data sources are presented in the Experimental Section. C,D) The survival rates of GBM patients with high and low expression of C) Sox2 and D) CD44. Sox2 and CD44 expression‐related GBM survival data were obtained from PrognoScan results. Data sources are presented in Materials and Methods. E,F) IHC staining of E) Sox2 and F) CD44 expression in human normal brain tissue and GBM tissue. Scale bar:100 µm. G,H) IHC staining of G) Sox2 and H) CD44 expression in mouse normal brain tissue and GBM tissue. Scale bar: 50 µm. I) IHC staining of Sox2 and CD44 expression in postoperative residual GBM tissues. Scale bar: 100 µm. Sox2 and CD44 stained IHC sections were derived from the same mouse GBM resection sample. J) Signal intensity of luciferase positive GL261 cells and GSCs mouse model after GBM establishment, surgical resection, and postoperative recurrence. Surgical resection was performed 12 days after GBM construction, and the recurrent fluorescent signals were observed 7 days after tumor resection. K) Quantitative analysis of signal intensity after GBM establishment, surgical resection, and postoperative recurrence from Figure 1J (*n* = 3). L) Survival rates of GL261 and GSCs bearing mice after surgical resection (*n* = 5). Data are expressed as mean ± S.D., *p < 0.05 (Student's *t*‐test).

We investigated the gene expression of SRY‐box‐containing gene 2 (Sox2) and CD44 indicative of GSCs in the Gene Expression Profiling Interactive Analysis (GEPIA) database. We found that both Sox2 and CD44 increased in human GBM tissues compared to normal brain tissue (**Figure** **1**A,B). We plotted survival curves for patients with high and low Sox2 and CD44 gene expression profiles using Kaplan‐Meier's method. 11 patients out of a sample of 70 GBM patients with an unknown time to endpoint event were recorded as right‐censored data (Figure S1, Supporting Information). Overexpression of Sox2 or CD44 significantly correlated with poor patient survival rates (Figure 1C,D). We performed IHC staining to investigate the expression of Sox2 and CD44 in tumor tissues. We found higher expression of Sox2 and CD44 in human GBM tissues compared to normal human brain tissue (Figure 1E,F). Similarly, Sox2 and CD44 also increased in mouse GBM tissue compared to normal brains (Figure 1G,H). These results demonstrated the presence of GSCs in GBM tumor tissues. We constructed mouse GBM resection models by implanting glioma 261 (GL261) cells in the mouse brain. After 12 days, the brain tumor was removed by surgical resection and performed with immunohistochemical (IHC) staining. We found that Sox2‐ and CD44‐positive GSCs remained present at unresected residual GBM tumor sites (Figure 1I). To demonstrate that the GSCs participate in GBM postoperative recurrence, we obtained GSCs by magnetic sorting from the GL261 cell line. Both flow cytometry analysis (Figure S2 in the Supporting Information) and immunofluorescent staining (Figure S3 in the Supporting Information) demonstrated the successful sorting and culture of GSCs. We constructed mouse GBM models with GL261 cells and GSCs, respectively. After 12 days of tumor cell implantation, the mouse GBM models derived from GSCs had higher tumor growth efficiency than those of GL261 cells. Subsequently, we performed surgical resection of the GBM and found the GSC‐derived mouse model exhibited stronger tumor fluorescence signals compared to that of GL261 cells after 7 days of the tumor recurrence (Figure 1J,K). In survival experiments, GSC‐derived GBM models also exhibited shorter survival times after surgical resection compared to GL261 cells (Figure 1L). All these results demonstrated that GSCs mediated the postoperative recurrence of GBM and promoted tumor secondary strikes.

### 2.2 Synthesis and Characterization of PMVS‐P

**Figure 2:**
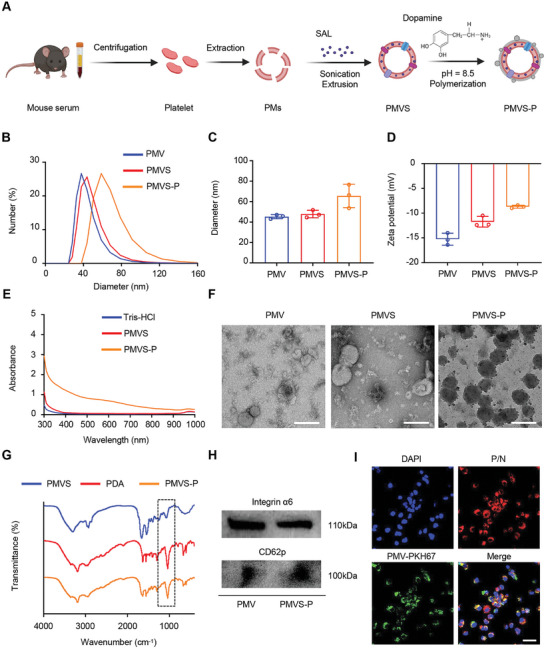
Synthesis and characterization of PMVS‐P. A) Schematic illustration of PMVS‐P synthesis. PMs were isolated from mouse blood by centrifugation. SAL was loaded in PMs by sonication and extrusion. PDA modification was performed by in situ polymerization of DA on the nanovesicle surface under the alkaline condition. B) Size distribution of PMV, PMVS, and PMVS‐P measured by DLS. C) Quantitative size analysis of PMV, PMVS, and PMVS‐P nanovesicles from Figure 2B. D) Zeta potential of PMV, PMVS, and PMVS‐P nanovesicles measured by DLS. E) Ultraviolet absorption spectrum of Tris‐HCl, PMVS, and PMVS‐P. F) TEM images of PMV, PMVS, and PMVS‐P. Scale bar: 100 nm. G) FTIR spectra of PMVS, PDA, and PMVS‐P nanomaterials. The absorption peaks resulting from the vibration of the C‐O bond of PDA are marked by black boxes. H) Analysis of integrin α6 and CD62p protein expression in PMV and PMVS‐P by western blotting. I) Intracellular co‐localization of PMV‐PKH67 and P/N after uptaking by GSCs. DAPI: nucleus (blue); PKH67: PMV (green); Nile red: PDA (red). Scale bar: 50 µm.

We isolated platelets from mouse blood by centrifugation and extracted platelet membranes (PMs). After that, SAL was loaded in PMs by sonication and several extrusion to form SAL‐encapsulating PM nanovesicles (PMVSs). PDA modification of PMVS surface (PMVS‐P) was performed by in situ polymerization of dopamine (DA) on the nanovesicle surface under the alkaline condition (**Figure** **2**A). PMV and PMVS showed good dispersion in optical photos and turned black after PDA modification with no precipitation or particle aggregation (Figure S4 in the Supporting Information). Dynamic light scattering (DLS) showed that the particle sizes of PMV, PMVS, and PMVS‐P were 45.3 ± 2.0, 47.9 ± 3.6, and 65.6 ± 11.5 nm, respectively (Figure 2B,C). The zeta potentials of PMV, PMVS, and PMVS‐P were −15.3 ± 1.2, −11.7 ± 1.1, and −8.7 ± 0.3 mV, respectively (Figure 2D). We continuously tested PMVS‐P by DLS for one week and found that its particle size and zeta potential had not changed significantly, proving the good stability of PMVS‐P (Figure S5A,B, Supporting Information). To calculate the loading efficiency, we obtained the standard curve of SAL by high‐performance liquid chromatography (HPLC). We found that adding 100 µg of SAL had the optimal loading efficiency of 10.8% ± 0.8%, corresponding to an encapsulation efficiency of 65.0% ± 4.8% (Figure S6–S9 in the Supporting Information) obtained by HPLC testing. We tested the obvious UV absorption spectra of PDA among 300–1000 nm, which was consistent well with the former reported^[^ ^11^ ^]^ (Figure S10 in the Supporting Information). Similar UV absorption spectra were also found in synthesized PMVS‐P compared to Tri‐HCl and PMVS, demonstrating the successful modification of PDA on the nanovesicles (Figure 2E). In situ PDA was observed on the surface of PMVS in the TEM image, which did not affect the dispersibility of the nanovesicle (Figure 2F). Since PDA is rich in C‐O chemical bonds and benzene rings, these two groups can be detected by Fourier transform infrared spectrometer (FTIR) and proton nuclear magnetic resonance ((1)H‐NMR), respectively, to determine that PDA is modified on PMVS.^[^ ^11b^ ^]^ In FTIR detection, PMVS‐P has an obvious C‐O bond vibration at 1100 cm^−1^, which is consistent with the vibrational peak of PDA (Figure 2G). In 1) H‐NMR assay, PMVS‐P has a distinct absorption peak at 6.5–7 ppm and is consistent with that of PDA, indicating that PMVS‐P carries a large number of benzene rings (Figure S11 in the Supporting Information). Thus, both experiments showed that PMVS‐P nanomaterials have the structural features of PDA, demonstrating the successful modification of PDA on PMVS. CD62p and integrin α6 are proteins that are stably expressed on platelet membranes and are involved in the physiological process of thrombosis.^[^ ^5^ ^]^ Western blotting showed that the expression of the key proteins in the PMVS‐P group was similar to that of PMV (Figure 2H), indicating that SAL encapsulation and PDA modification did not affect the protein expression in platelet membrane vesicles. PDA has strong adhesive properties and can be functionalized with fluorescent probes on the surface for molecular tracking.^[^ ^12^ ^]^ The phospholipid bilayer of cell membranes can also be tagged by lipophilic dyes.^[^ ^13^ ^]^ Therefore, the successful binding of PDA to PMV can be demonstrated by the co‐localization of two different fluorescent probes. Here, we modified the phospholipid layer of the PMV and PDA layer with PKH‐67 and Nile Red (NR), respectively. The nanovesicles exhibited co‐localization of PKH‐67 with NR in a confocal laser scanning microscope (CLSM) after being uptaken by GSCs, also indicating the successful integration of PMV with PDA (Figure 2I).

### 2.3 In Vitro Tumor Cytotoxicity of PMVS‐P

**Figure 3:**
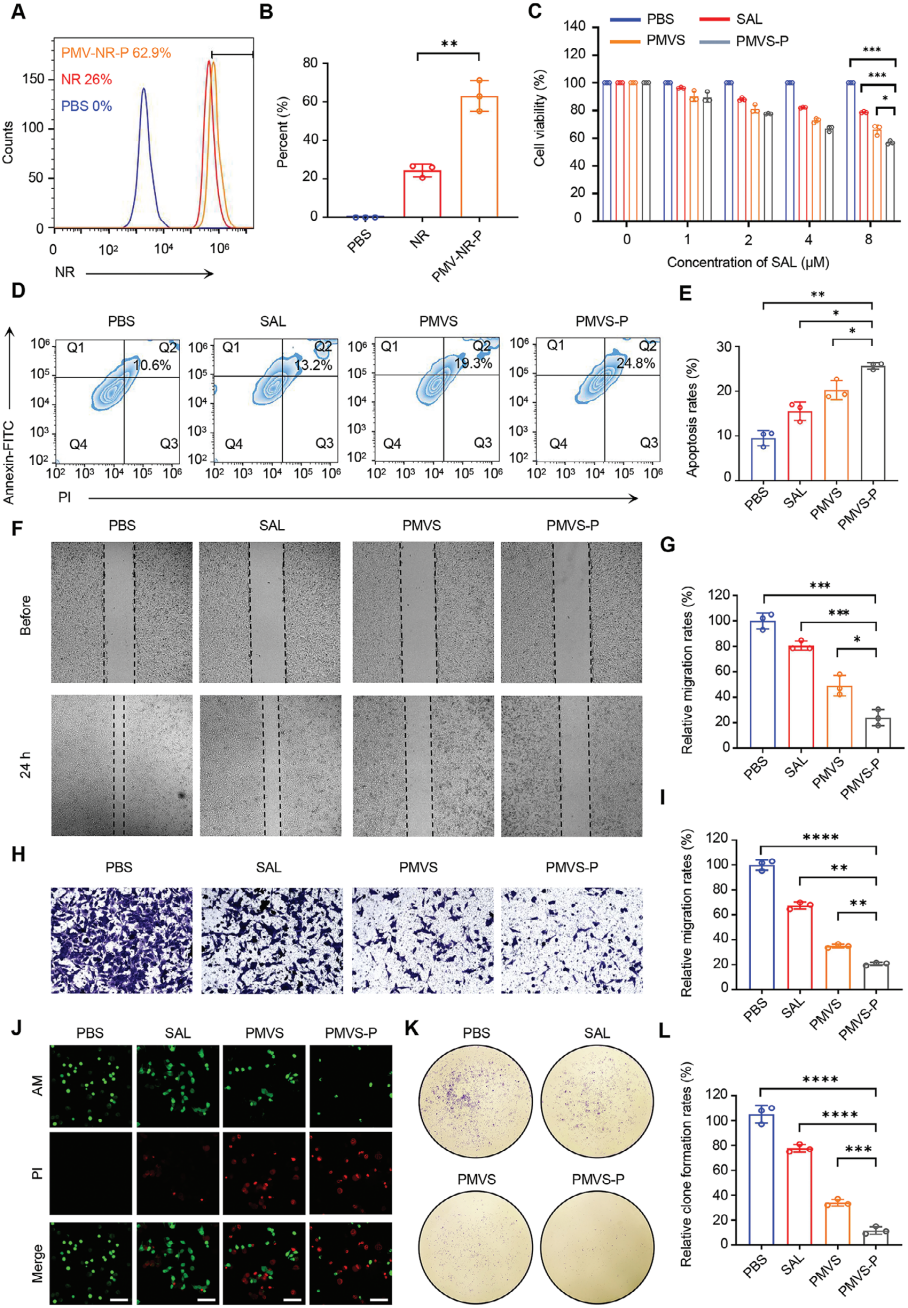
Tumor cytotoxic effects of PMVS‐P. A) Flow cytometry analysis of the cellular uptake efficiency of PBS, NR, and PMV‐NR‐P. Different formulations were incubated with GL261 cells for 2 h. B) Quantitative analysis of cellular uptake efficiency of PBS, NR, and PMV‐NR‐P from Figure 3A. C) Viability of GL261 cells after treatment with different formulations (PBS, SAL, PMVS, and PMVS‐P) with varied SAL concentrations. Cell viability was evaluated by cell‐counting kit 8 (CCK8) assay. D) Apoptosis rates of GL261 cells after being treated with different formulations (PBS, SAL, PMVS, and PMVS‐P). The cells were stained with Annexin‐FITC/PI Kit and apoptosis rates were tested by flow cytometry. E) Quantitative analysis of apoptosis rates from Figure 3D. F) Wound healing assay showing the migration of GL261 after treatment with PBS, SAL, PMVS, and PMVS‐P for 24 h. G) Quantification of relative migration rates of G261 cells after PBS, SAL, PMVS, and PMVS‐P treatment from Figure 3F. H) GL261 cell migration in transwell model after 24 h after treatment of PBS, SAL, PMVS, and PMVS‐P. I) Quantification of relative migration rates of G261 cells after PBS, SAL, PMVS, and PMVS‐P treatment from Figure 3H. J) CLSM image showing Calcein‐AM/PI staining of GL261 cells after being treated with PBS, SAL, PMVS, and PMVS‐P for 48 h. Calcein‐AM: green; PI: red. Scale bar: 100 µm. K) Representative images of tumor clone clusters after PBS, SAL, PMVS, and PMVS‐P treatment. L) Relative rates of tumor clone formation analyzed from Figure 3K (*n* = 3). Data are expressed as mean ± S.D., *p < 0.05; **p < 0.01; ***p < 0.001; ****p < 0.0001 (Student's *t*‐test).

We further investigated the cellular uptake efficiency of the nanovesicles by GL261 cells. NR‐labeled nanovesicles (PMV‐NR‐Ps) were incubated with GL261 cells for 2 h, then the NR fluorescent intensity in GL261 cells was tested by flow cytometry, which is indicative of cellular uptake of the nanovesicles. The cellular uptake efficiency of PMV‐NR‐P was determined to be 63.1% ± 8.0%, significantly higher than that of NR (24.4% ± 3.3%) (**Figure** **3**A,B). The cytotoxicity of the nanovesicles to GL261 cells was investigated by exploring the viability of GL261 cells treated with different formulations at varied concentrations of SAL. Cell counting kit‐8 (CCK‐8) assay showed decreased cell viability with increasing SAL concentration, indicating the anti‐tumor effect of SAL. PMVS‐P group exhibited lower cell viability (56.9% ± 1.2%) compared to SAL group (78.8% ± 0.4%) and PMVS group (66.2% ± 3.5%), indicating that PMV encapsulation and PDA modification enhanced tumor toxicity of SAL (Figure 3C). We further tested the apoptotic effect of PMVS‐P on GBM since salinomycin produces tumor cytotoxicity mainly dependent on the apoptotic pathway.^[^ ^14^ ^]^ The apoptosis rate was higher in the PMVS‐P group (25.6% ± 0.7%) than in the SAL group (15.5% ± 2.0%) and PMVS group (20.3% ± 2.1%) (Figure 3D,E), indicating that PM encapsulation and PDA modification promoted the apoptotic effect of SAL. Tumor malignancy is closely related to its migration ability, so we tested the inhibitory property of PMVS‐P on tumor cell migration. Wound healing assay showed that PMVS‐P‐treated GL261 cells had a significantly lower migration rate (24.0% ± 6.4%) compared to the SAL (80.6% ± 3.6%) and PMVS (49.2% ± 8.1%) group (Figure 3F,G). In transwell assay, the migration rate of PMBS‐P‐treated GL261 cells was significantly lower (20.7% ± 1.2%) than that of SAL (67.5% ± 2.8%) and PMVS (35.0% ± 1.5%) (Figure 3H,I). These results demonstrated the inhibited tumor cell migration by PMVS‐P. CLSM images revealed that PMVS‐P significantly increased PI‐positive dead cells and decreased calcein‐AM‐positive live cells compared to PBS, SAL, and PMVS formulations (Figure 3J), indicating that PM and PDA modifications increase the lethality of SAL on tumor cells. Tumor tissue is formed by the clonal proliferation of single or multiple progenitor tumor cells, a process that reflects the malignancy of the tumor.^[^ ^15^ ^]^ Therefore, we diluted tumor cells in vitro and performed single‐cell cloning experiments. We found that PMVS‐P (11.7% ± 3.1%) significantly inhibited clonal cluster formation from GBM monocytes compared to SAL (77.7% ± 3.1%) and PMVS (34.0% ± 2.6%), respectively (Figure 3K,L), indicating that PM and PDA modifications promoted the inhibition of SAL on the proliferative efficiency of tumor clusters. Taken together, these results suggested that PMVS‐P can restrain the malignant proliferation of GBM by inhibiting tumor cell apoptosis, necroptosis, migration, and clone formation. Studies have shown that tumor stem cells play an important role in maintaining both malignant proliferation and migration of tumors.^[^ ^16^ ^]^ SAL is a small molecule inhibitor with tumor stem cell toxicity.^[^ ^17^ ^]^ The promotion of SAL cytotoxicity by PMV and PDA modification may be due to the enhanced targeting of PMVS‐P to tumor stem cells. We therefore intended to investigate the targeting and toxicity of PMVS‐P to the tumor stem cells.

### 2.4 D2DR‐Mediated GSC Targeting and Inhibition by PMVS‐P

**Figure 4:**
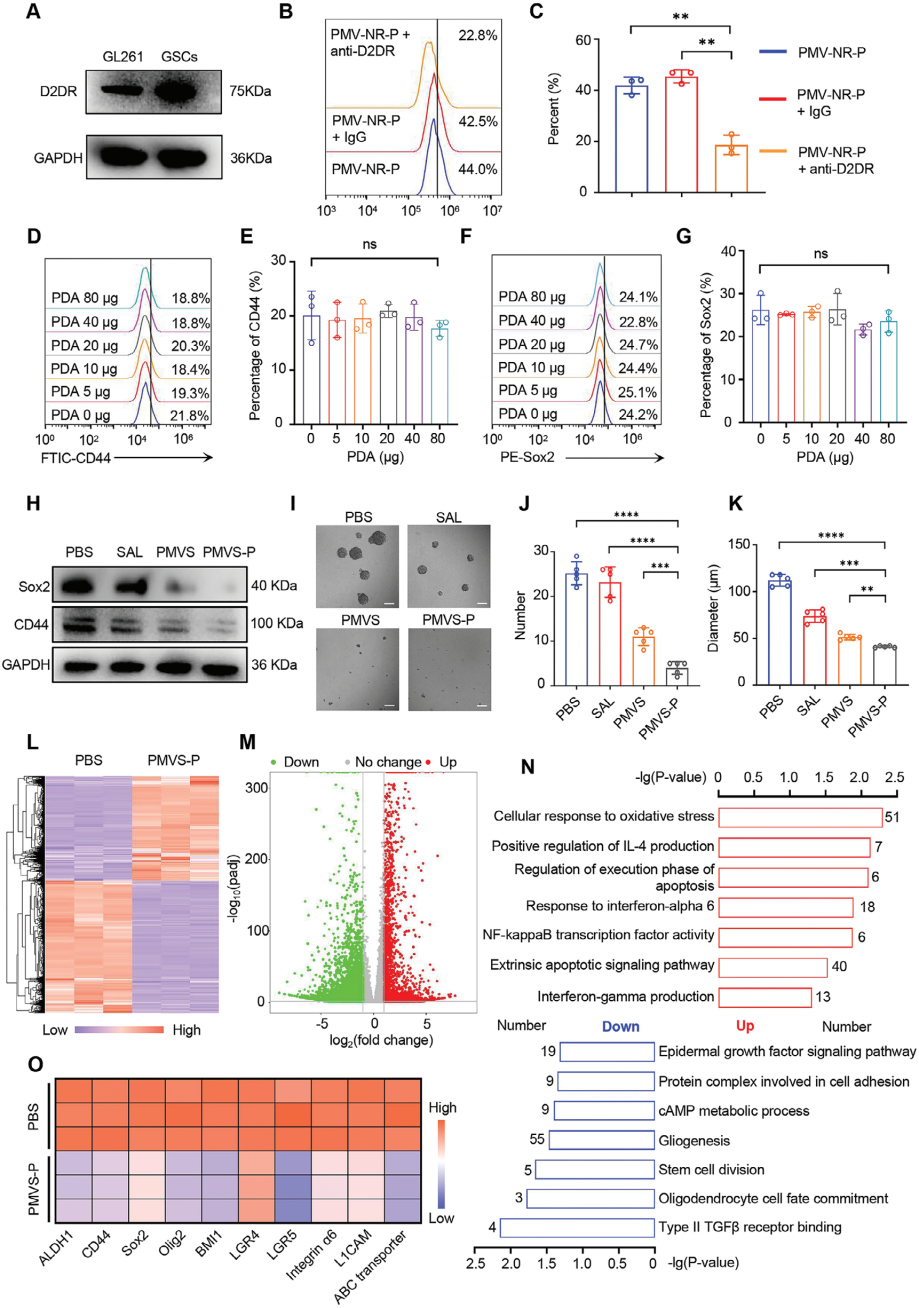
D2DR‐mediated nanoparticle uptake of GSCs and gene expression profiling detection after PMVS‐P treatment. A) D2DR expression in GL261 cell line and GSCs tested by western blotting. B) Representative images of cellular uptake efficiency in GSCs after treatment with different formulations (PMV‐NR‐P, PMV‐NR‐P + IgG, and PMV‐NR‐P + anti‐D2DR). C) Quantitative analysis of cellular uptake efficiency in GSCs from Figure 4B (*n* = 3). D) Representative images of CD44 expression in GL261 cells after treatment with different masses of PDA tested by flow cytometry. E) Quantitative analysis of CD44 expression in GL261 cells after treatment with different masses of PDA from Figure 4D (*n* = 3). F) Representative images of Sox2 expression in GL261 cells after treatment with different masses of PDA tested by flow cytometry. G) Quantitative analysis of Sox2 expression in GL261 cells after treatment with different masses of PDA from Figure 4F (*n* = 3). H) Sox2 and CD44 expression in GSCs after treatment with PBS, SAL, PMVS, and PMVS‐P. I) Representative bright field images of GSC spheres after being treated with PBS, SAL, PMVS, and PMVS‐P. Scale bar: 100 µm. J,K) Quantitative analysis of J) number and K) size of GSC spheres after being treated with PBS, SAL, PMVS, and PMVS‐P from Figure 4I (*n* = 5). L) The heat map of the hierarchical clustering of differentially expressed genes after PMVS‐P treatment compared to the PBS group. GL261 cells were used to detect the gene expression profile. M) Volcano plots showed the up‐and down‐regulated genes after PMVS‐P treatment compared to the PBS group. N) GO enrichment analysis of differentially expressed genes after PMVS‐P treatment compared to the PBS group. O) GSCs‐related potential gene expression after PMVS‐P treatment (*n* = 3). Data are expressed as mean ± S.D., ns, no significant; *p < 0.05; **p < 0.01; ***p < 0.001; ****p < 0.0001 (Student's *t*‐test).

We obtained GSCs from the GL261 cell line by magnetic sorting. Flow cytometry analysis showed that the expression of CD133 in GSCs increased from 7.8% to 74.3% compared to GL261 cells (Figure S2 in the Supporting Information). After culturing in stem cell medium, the expression of cancer stem cell markers such as Nanog, Sox2, Nestin, and oligodendrocyte transcription factor 2 (Olig2) in GSC spheres was confirmed by immunostaining (Figure S3 in the Supporting Information). These results demonstrated the successful isolation and culture of GSCs. We found that the expression of D2DR was significantly higher in GSCs than in bulk GL261 tumor cells (**Figure** **4**A). D2DR can be identified by PDA through catechol and amine groups and mediate endocytosis of PDA‐modified nanoparticles.^[^ ^10^ ^]^ Therefore, we examined the efficiency of PDA‐guided nanovesicle uptake by GSCs. We used IgG as a control to exclude nonspecific adsorption of antibodies. The uptake rates in GSCs were 41.9% ± 3.3% and 45.4% ± 2.5% for the PMVS‐P and PMVS‐P + IgG groups, respectively, which were both significantly reduced by anti‐D2DR (18.6% ± 3.8%), demonstrating D2DR‐mediated endocytosis of PMVS‐P in GSCs (Figure 4B,C). Since some studies have shown that D2DR activation promoted the restoration of GSCs phenotype, which increased tumor malignancy,^[^ ^8a^ ^]^ we investigated the effect of PDA on the restoration of GSCs. We found that a large dose (80 µg) of PDA, a dosage far exceeding PDA in PVMS‐P used in the cellular experiments, had no significant effect on either Sox2 or CD44 expression (Figure 4D–G). These results indicated that D2DR promoted PDA‐mediated endocytosis in GSCs without affecting their phenotype. Similar to the previous studies, this phenomenon was related to the lost bioactivity of dopamine caused by changes in the chemical structure during the polymerization reaction.^[^ ^18^ ^]^ Subsequently, we verified the toxic effects of PMVS‐P on GSCs. Western blotting showed the inhibition of Sox2 and CD44 expression in GSCs by SAL treatment, which was significantly enhanced after PMV encapsulation (PMVS). PMVS‐P exhibited the most apparent inhibition effect (Figure 4H). Flow cytometry analysis also demonstrated the reduced expression of cancer stem cell markers CD15 and CD133 in GSCs after PMVS‐P treatment (Figures S12 and S13 in the Supporting Information). These results indicated the improved GSC inhibition effect of PMVS‐P. We performed GSC spheronization experiments to verify the growth inhibition of PMVS‐P on tumor stem cell spheres. Similar to the trend observed with GSC marker expression, the number and size of GSC tumor spheres were reduced by SAL treatment (number: 25.2 ± 2.6 and size: 112.2 ± 6.3 µm in PBS group; number: 23.2 ± 3.4 and size: 73.8 ± 6.6 µm in SAL treatment group). The inhibition effect of SAL on tumorsphere growth was significantly improved by platelet‐derived nanovesicle encapsulation (number: 11.0 ± 2.0 and size: 51.4 ± 3.0 µm in PMVS group) and PDA modification (number: 4.0 ± 1.4 and size: 41.0 ± 1.0 µm in PMVS group) (Figure 4I–K). These results, taken together with the increased cellular uptake mediated via D2DR, demonstrated PDA promoted the GSC targeting and the inhibition effect of PMVS‐P.

We further detected the gene expression profile of PMVS‐P‐treated GBM cells compared to the PBS group. The reads mapped to genome regions in PBS and PMVS‐P groups are shown in Figure S14, (Supporting Information). A huge number of differentially expressed genes (DEGs) were detected in the PMVS‐P group compared to the PBS group (Figure 4L). Volcano plots identified 2362 upregulated (red) and 3735 downregulated (green) genes after PMVS‐P treatment (Figure 4M). Gene set enrichment analysis (GSEA) showed that the cellular component, signaling pathway, and molecular function changed in Figure S15 (Supporting Information). Gene ontology (GO) analysis showed that down‐regulated genes were associated with cancer stem cell division, cell adhesion, growth factor pathway, and gliogenesis, while up‐regulated genes were related to oxidative stress, inflammatory factors, and apoptotic pathways (Figure 4N). To identify the altered GSCs genes in response to PMVS‐P treatment, the correlated cancer stem cell genes were analyzed from the gene database. Our results showed that ALDH1, CD44, Sox2, Olig2, BMI1, LGR4, LGR5, Integrin α6, L1CAM, and ABC transporter associated with cancer stem cells were significantly downregulated (Figure 4O). These results further demonstrated the inhibitory effect and therapeutic potential of PMVS‐P on GSCs.

### 2.5 BBB Penetration and Postoperative Residual GBM Tissue Targeting of PMVS‐P

**Figure 5:**
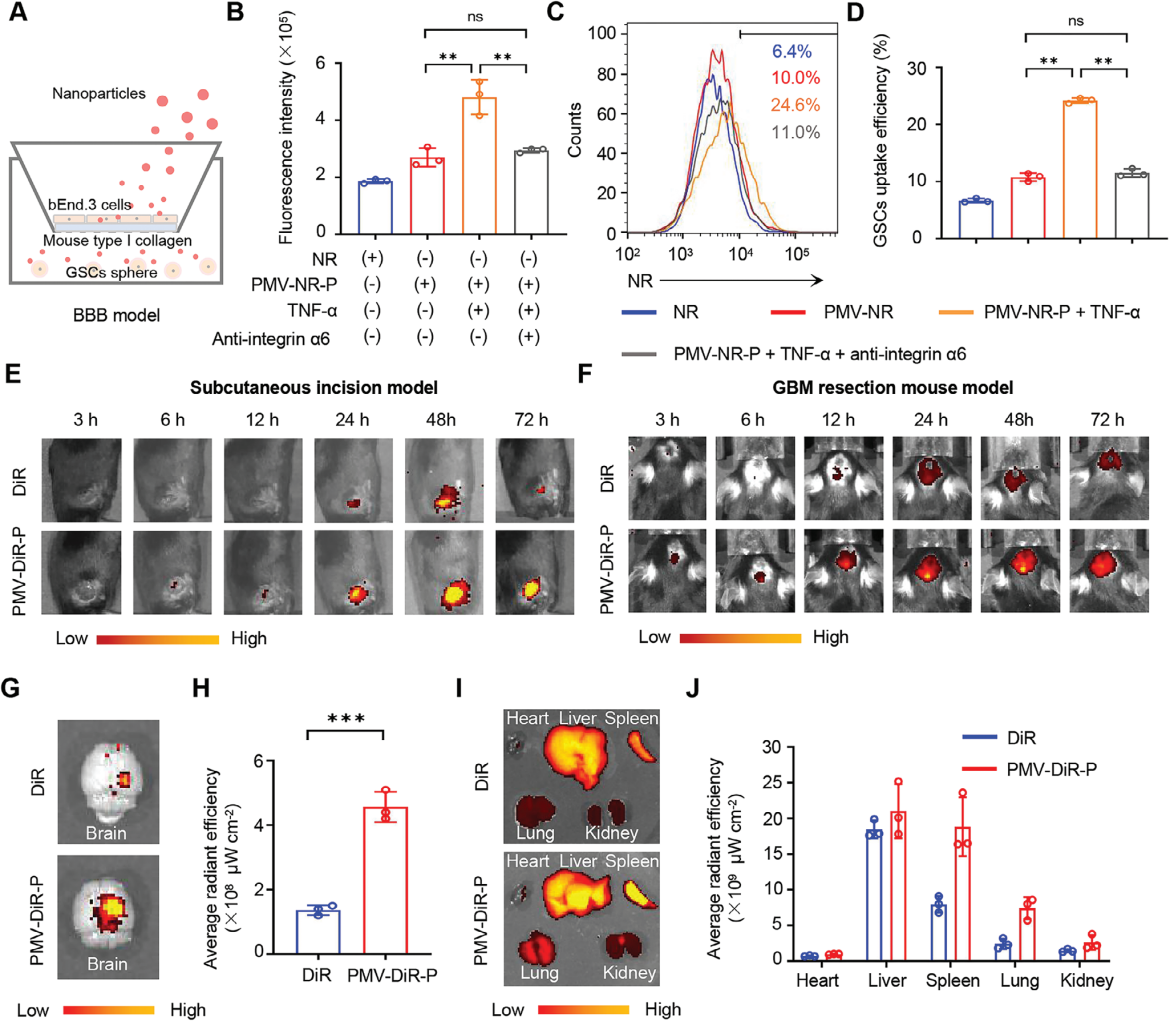
BBB crossing and postoperative incision targeting. A) An in vitro BBB model was constructed using a 0.4 µm pore size transwell with brain microvascular endothelial cell line bEnd.3 and mouse type I collagen in the upper chamber, and GSC spheres in the lower chamber. To mimic inflammation‐mediated BBB injury, 15 ng mL^−1^ TNF was added and pretreated for 24 h. 2 µg of integrin α6 antibody was pretreated with 100 µg PMV‐NR‐P for 2 h. B) NR signal intensity in the lower chamber after treatment with different formulations for 8 h. C) Representative images of NR and PMV‐NR‐P uptake efficiency by GSC spheres in the lower chamber after treatment with different formulations for 8 h tested by flow cytometry. D) Quantitative analysis of NR and PMV‐NR‐P uptake efficiency by GSC spheres from Figure 5C. E) Signal intensity of DiR and PMV‐DiR‐P in subcutaneous incisions at different time points detected by in vivo imaging system (IVIS). F) Signal intensity of DiR and PMV‐DiR‐P in GBM surgical incisions at different time points detected by IVIS. G) Signal intensity of DiR and PMV‐DiR‐P in postoperative brain tissue at 48 h after injection. H) Quantitative analysis of DiR and PMV‐DiR‐P in postoperative brain tissue at 48 h after injection from Figure 5G (*n* = 3). I) Representative images of the signal intensity distribution of DiR and PMV‐DiR‐P in major metabolic organs after 48 h treatment (*n* = 3). J) Quantification analysis of DIR and PMV‐DiR‐P signal intensity in major metabolic organs from Figure 5I. Data are expressed as mean ± S.D., *p < 0.05; ***p < 0.001 (Student's *t*‐test).

Engineered cell membrane nanovesicles facilitate nanoparticles across the BBB and contribute to drug delivery into the brain parenchyma.^[^ ^19^ ^]^ PMV facilitates BBB crossing by binding expressed integrins to the exposed subendothelial collagen at the site of brain injury.^[^ ^4b^ ^]^ Inflammatory factors (e.g., TNF‐α) produced during brain surgery lead to the destruction of vascular endothelial cells and collagen exposure, thus promoting BBB crossing of PMVs.^[^ ^20^ ^]^ We constructed an in vitro BBB model using a transwell chamber, added TNF‐α to mimic the inflammatory environment of the brain and tested the mechanism of PMVS‐P crossing the BBB (**Figure** **5**A). We found that PMV‐NR‐P had higher fluorescence intensity (4.8 × 10^5^ ± 0.6 × 10^5^) in the lower chamber after TNF‐α pretreatment of the BBB model compared with NR (1.9 × 10^5^ ± 0.07 × 10^5^) and PMV‐NR‐P (2.7 × 10^5^ ± 0.3 × 10^5^) groups, which was reduced after nanovesicles were pretreated with anti‐integrin α6 antibody (2.9 × 10^5^ ± 0.08 × 10^5^) (Figure 5B). Similarly, flow cytometry analysis showed that cellular uptake of PMV‐NR‐P in GSCs in the lower chamber had significantly increased fluorescence intensity (24.2% ± 0.5%) after TNF‐α pretreatment compared with NR (6.7% ± 0.4%) and PMV‐NR‐P (10.8% ± 0.7%) groups. Besides, this efficiency was also reduced after nanovesicles were pretreated with anti‐integrin α6 antibody (11.5% ± 0.7%) (Figure 5C,D). All these results indicated that PMV binds to the inflammation‐damaged BBB via integrin α6 and thus improves crossing efficiency. Platelet‐derived vesicles are endowed with wound‐targeting properties,^[^ ^21^ ^]^ and hence have great potential in drug delivery for inhibiting tumor recurrence after surgery. To demonstrate platelet membrane‐mediated surgical incision targeting, we constructed a subcutaneous incision model. We found that the PMV‐DiR‐P signal was significantly higher than DiR, reached the peak at 24 – 48 h, and gradually weakened after 72 h, indicating the successful targeting of PMVS‐P at wound sites (Figure 5E). Similarly, in the GBM model of surgical resection, the PMV‐DiR‐P signal also reached the peak at 24 – 48 h and was significantly higher than DiR, demonstrating the successful targeting of PMVS‐P to GBM surgical incisions (Figure 5F). Subsequently, we dissected the brain and vital organs of the mice 48 h after injection of the nanoparticles. PMV‐DiR‐P‐treated nice (4.6 × 10^8^± 0.5 × 10^8^µW cm^−^) showed significantly higher signal intensity than the DiR group (1.4 × 10^8^ ± 0.2 × 10^8^ µW cm^−^) in postoperative GBM tissues (Figure 5G,H), which was metabolized mainly in liver, spleen and kidney organs (Figure 5I,J). These results demonstrated that PMV loading significantly increased the aggregation of nanoparticles at the postoperative GBM site.

### 2.6 Postoperative Recurrence Inhibition of GBM by PMVS‐P In vivo

**Figure 6:**
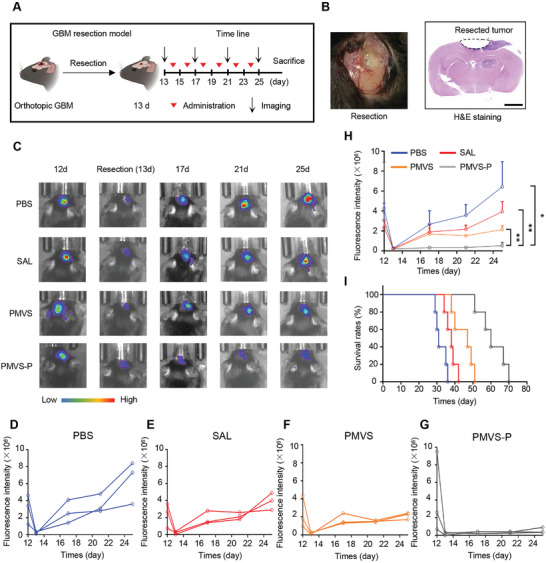
GBM postoperative recurrence inhibition effect of PMVS‐P in vivo. A) The time points of surgical resection, drug injection, imaging, and sacrifice of GBM‐bearing mice. B) GBM surgical resection optical image and H&E staining. Scale bar: 2 mm. C) Representative images of GBM postoperative recurrence signal intensity after PBS, SAL, PMVS, and PMVS‐P treatments. D–G) The individual GBM recurrence signal intensity after D) PBS, E) SAL, F) PMVS, and G) PMVS‐P treatments. H) The average GBM recurrence signal intensity after PBS, SAL, PMVS, and PMVS‐P treatments (*n* = 3). I) The survival rates curve of GBM resection mice after PBS, SAL, PMVS, and PMVS‐P treatments (*n* = 5). Data are expressed as mean ± S.D., *p < 0.05; **p < 0.01 (Student's *t*‐test).

**Figure 7:**
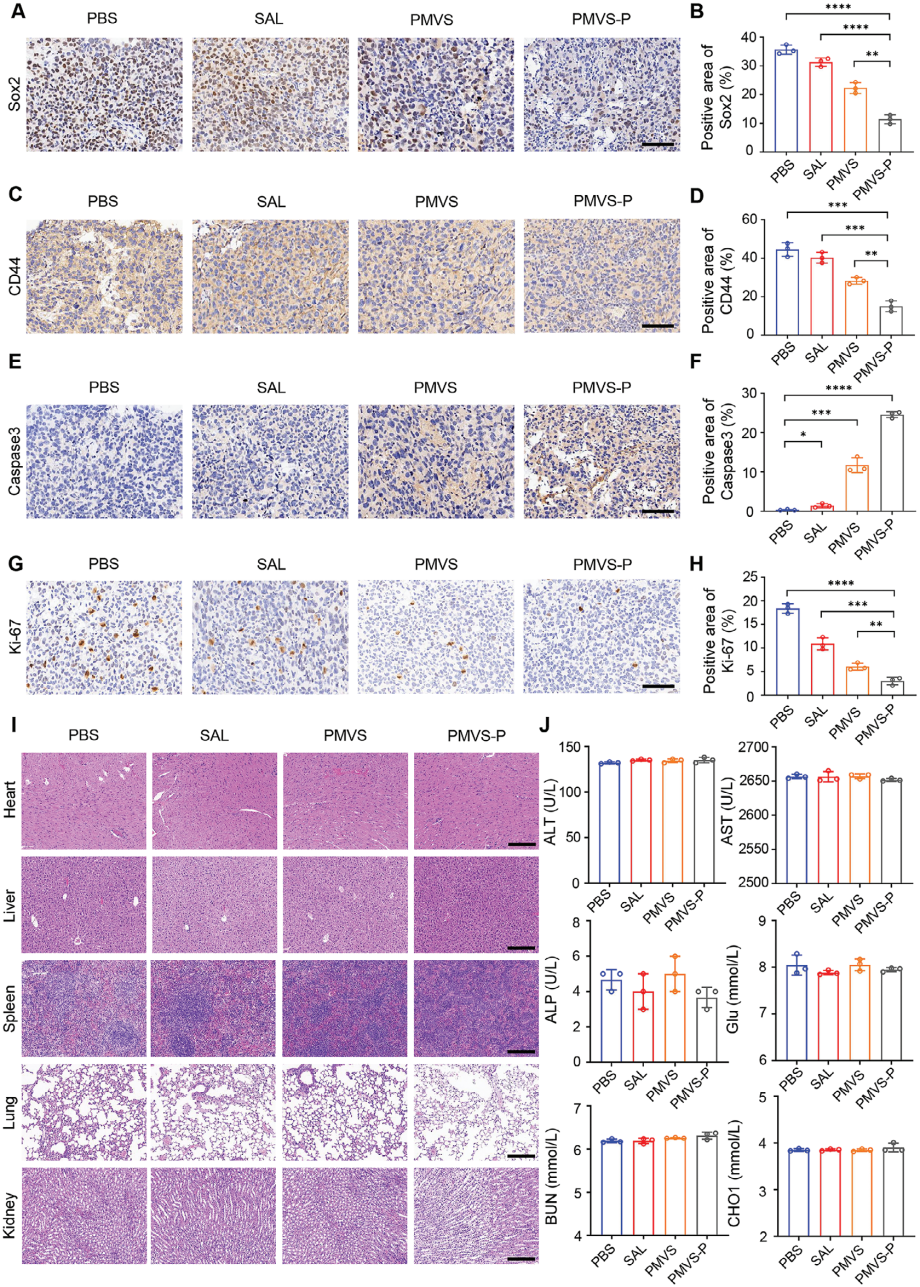
Cancer stem cell inhibitory effect and biotoxicity assessment in vivo after PMVS‐P treatment. A) The representative images of IHC staining for Sox2 expression. Scale bar: 100 µm. B) The quantitative analysis of Sox2 expression from Figure 7A (*n* = 3). C) The representative images of IHC staining for CD44 expression. Scale bar: 100 µm. D) The quantitative analysis of CD44 expression from Figure 7C (*n* = 3). E) The representative images of IHC staining for Caspase3 expression. Scale bar: 100 µm. F) The quantitative analysis of caspase3 expression from Figure 7E (*n* = 3). G) The representative images of IHC staining for Ki‐67 expression. Scale bar: 100 µm. H) The quantitative analysis of Ki‐67 expression from Figure 7G (*n* = 3). I) H&E staining of heart, liver, spleen, lung, and kidneys after PBS, SAL, PMVS, PMVS‐P treatment. Scale bar: 200 µm. J) Concentrations of ALT, AST, ALP, Glu, BUN, and CHO1 in serum after PBS, SAL, PMVS, and PMVS‐P treatment (*n* = 3). Data are expressed as mean ± S.D., *p < 0.05; **p < 0.01; ***p < 0.001; ****p < 0.0001 (Student's *t*‐test).

To verify the inhibitory effect of PMVS‐P on GBM recurrence after surgery in vivo, we constructed an orthotopic GBM model and performed surgical resection on day 13 (**Figure** **6**A). Optical image and H&E staining showed successful partial resection of GBM (Figure 6B). Biofluorescence intensity is proportional to the amount of Luc‐GL261 cells, so we used in vivo tumor fluorescence intensity quantification to determine GBM size (Figure S16 in the Supporting Information). After different formulations of treatment, PMVS‐P exhibited strong GBM recurrence inhibition ability compared to the PBS, SAL, and PMVS groups (Figure 6C). The individual and average signal intensity of GBM had similar results (Figure 6D–H). PMVS‐P also significantly prolonged the survival of mice after GBM resection compared to the PBS, SAL, and PMVS groups (Figure 6I). The mean, variance, and standard deviation of the fluorescence intensity of the tumor recurrence after surgical resection in the PBS, SAL, PMVS, and PMVS‐P groups at different time points are shown in Table S1 (Supporting Information). Mouse body weights did not change significantly over the survival period, suggesting the low toxicity of PMVS‐P (Figure S17 in the Supporting Information). IHC staining and quantification showed that the expression of Sox2 was significantly lower in the PMVS‐P treatment group (11.4% ± 1.6%) than in the PBS (35.6% ± 1.6%), SAL (31.3% ± 1.4%) and PMVS (22.3% ± 1.9%) group (**Figure** **7**A,B). The expression of CD44 in the PMVS‐P treatment group (15.0% ± 2.8%) was similarly significantly lower than that in the PBS (44.6% ± 3.5%), SAL (40.3% ± 2.8%) and PMVS (28.3% ± 1.8%) groups (Figure 7C,D). These results indicated that the modification of PDA promoted the toxicity of nanoparticles to GSCs. In addition, PMVS‐P (24.6% ± 0.8%) also increased the rates of apoptosis in GBM cells compared to the PBS (0.4% ± 0.1%), SAL (1.4% ± 0.5%), and PMVS (11.7% ± 1.9%) groups (Figure 7E,F). The proliferation marker Ki‐67 decreased in PMVS‐P group (3.0% ± 0.8%) compared to PBS (18.4% ± 1.0%), SAL (10.9% ± 1.3%) and PMVS (6.1% ± 0.7%) groups (Figure 7G,H). These results suggested that PMVS‐P inhibited growth and enhanced apoptosis of GBM cells.

To examine the effects of PMVS‐P on organ microstructure in mice, we dissected the hearts, livers, spleens, lungs, and kidneys of GBM‐bearing mice after treatment. H&E staining revealed the structural integrity of each organ (Figure 7I). We measured ALT, AST, and ALP for liver function, Glu for blood glucose, BUN for kidney function, and CHO for blood lipids. The results showed that there were no significant changes in the serum of PMVS‐P‐treated mice compared to the PBS group (Figure 7J). These results demonstrated the low biotoxicity and high biocompatibility of PMVS‐P.

## 3 Discussion

Recurrence of GBM at the margin of the surgical incision remains poor quality of survival and lack of aggressive multimodal effective treatments, which is mainly associated with self‐renewing GSCs.^[^ ^3^ ^]^ Effectively inhibiting the residual GSCs is challenging due to the lack of highly biocompatible biomaterials for surgical incision and GSCs targeting. We demonstrated that GSCs highly express the neurotransmitter receptor D2DR (Figure 4A), which is specific for dopamine in the central nervous system. Our results showed that PMVS‐P neurotransmitter‐mimicking nanovesicle promoted endocytosis of GSCs through PDA‐mediated D2DR identification, thus exacerbating the toxicity of GSCs and inhibiting GBM recurrence. Moreover, the low immunogenicity of PMVS‐P also protects the nanovesicles from being cleared by the immune system,^[^ ^6^ ^]^ thus reducing immune rejection of PMVS‐P at the incision site. As a bioautogenous material, PMVS‐P also has high biocompatibility and low biotoxicity and does not cause significant side effects on the organs of mice (Figure 7I,J).

Although research has shown that the photothermal effect of PDA‐based nanoparticles had the potential to inhibit cancer stem cells without modifying any targeted peptides or proteins, the mechanism has not yet been elucidated.^[^ ^22^ ^]^ Our results shed light on the main mechanism of PDA‐based nanomaterials‐mediated cancer stem cell‐specific treatment. The synthesized neurotransmitter‐mimicking nanovesicle successfully inhibited the expression of cancer stem cell markers such as CD44 and Sox2 by D2DR identification (Figure 4A–C,H. Moreover, D2DR is not only distributed in central nervous system tumors but also in other tumors with endocrine functions (e.g., breast,^[^ ^23^ ^]^ kidney,^[^ ^24^ ^]^ lung,^[^ ^25^ ^]^ and colorectal cancers^[^ ^26^ ^]^) and has been used as a new target for cancer stem cell therapy.^[^ ^8c^ ^]^ Therefore, PMVS‐P nanomaterials can also be similarly extended for use in cancer stem cell‐specific tumoricidal therapy of these tumors, which is a significant direction for future research.

GSCs therapy has long been considered an effective method for inhibiting malignant tumor growth and postoperative recurrence. However, the microenvironment of GSCs and their location within tumors limits the design and development of GSCs‐targeting materials. In our work, we successfully achieved the inhibition of GSCs by modifying PDA nanomaterials on the surface of PMVS and provided a new platform for targeted drug delivery to GSCs. Furthermore, PDA is a widely used biomaterial and has been directly involved in drug delivery to tumors as a drug delivery nanocarrier or nanomaterial coating. Given the targeting of D2DR by PDA, our study also provides a new biological function for this type of nanomaterials for the targeting of GSCs.

In summary, we synthesized PMVS‐P nanomaterials by loading the anti‐GSCs drug SAL on PMVs and modifying PDA on the surface. PMVS‐P nanomaterials possessed GBM surgical incision targeting ability and recognized residual GSCs via D2DR receptors, successfully inhibiting GBM recurrence. Our results provided a new therapeutic strategy for the widely used PDA‐based nanomaterials for tumor recurrence.

## 4 Experimental Section

### Cell Lines, Materials, and Human Samples

GL261, Luc‐GL261, bEnd.3 cell lines were obtained from the National Center for Nanoscience and Technology. Dopamine hydrochloride (Maclin), NaOH (Macklin), D‐luciferin sodium (ANDY, 103404‐75‐7), DiR (Sigma‐Aldrich), DMEM/F12 (Gibco), Fetal Bovine Serum (Gibco), Penicillin‐Streptomycin (Gibco), Nile red (HY‐D0718), PKH‐67 (Umibio, UR52303), Salinomycin (MCE), anti‐CD44 (Proteintech, 15675‐1‐AP), anti‐Sox2 (CST, 23064s), anti‐GAPDH (proteintech, 60004‐1‐Ig). The patient sample was approved by First Affiliated Hospital of Chongqing Medical University (2017‐032). Sox2 and CD44 gene expression platforms in patients with GBM and healthy people were retrieved from The Cancer Genome Atlas (http://gepia.cancer‐pku.cn/detail.php?gene = &clicktag = boxplot). Sox2 and CD44 expression‐related GBM survival data were obtained from PrognoScan results (http://dna00.bio.kyutech.ac.jp/PrognoScan/index.html). In the Sox2 and CD44 gene expression‐associated survival assessment, the time to the endpoint event was unknown in 11 out of the 70 GBM patients. Data from these patients were recorded as right‐censored data.

### PMVS‐P Preparation

According to a previous study, platelet membranes (PMs) were extracted and synthesized from C57 mouse serum.^[^ ^5^ ^]^ Briefly, C57 mice were subjected to orbital blood collection. The blood was rested at 4 °C for 1 h and then centrifuged with 200 g for 20 min to obtain platelet‐rich plasma. Afterward, platelet‐rich plasma was centrifuged at 800 g for 20 min to obtain PMs. Platelets were frozen at −80 °C and warmed at 37 °C for five cycles. The PMs were precipitated by 12 000 g centrifugation for 30 min at 4 °C and resuspended by 10 mm Tris‐HCl for further use. For PMVS synthesis, different concentrations of SAL (50, 100, 200, and 400 µg) were added to 1 mg of PMs (2 µg µL^−1^) and sonicated for 10 min. Then, the mixture was passed through 800, 400, 200, and 100 nm filter membranes and continuously extruded. For PMVS‐P synthesis, 1 mL PMVS was added with 500 µg of dopamine, followed by adjusting the pH to 8.5 with NaOH and stirring for 2 h at room temperature. The synthesized PMVS‐P was washed 2 times by ultrafiltration. NR or DiR‐loaded nanoparticles were obtained by replacing SAL with NR or DiR by the method described above. The particle size and zeta potential of nanoparticles were obtained by DLS detection. Encapsulation efficiency and loading efficiency of SAL were detected by high‐performance liquid chromatography (HPLC). Briefly, different masses of SAL (1, 2, 3, and 4 µg) were added and the absorbance at 192 nm was measured by HPLC. The standard curve of SAL was obtained by linearly fitting the ratio of area under the curve to mass. Subsequently, the mass of SAL unloaded with nanoparticles in the cleaning solution was calculated from the standard curve. The encapsulation efficiency of SAL = (T_SAL_ – U_SAL_) / T_SAL_, and the loading efficiency of SAL = (T_SAL_ – U_SAL_) / (T_PMV_ + T_PDA_ + T_SAL_). The T and U represent the mass of total and unloaded drugs, respectively.

### Cellular Uptake and Cytotoxicity of PMVS‐P

GL261 cells were seeded in a 6‐well plate (5 × 10^5^ cells per well). PBS, NR, and PMV‐NR‐P (5 µm NR) were added for 2 h incubation. Cells were fixed in 4% paraformaldehyde after digestion by trypsin and subsequently used for flow cytometry analysis.

The cytotoxic effects of PMVS‐P were verified by cell counting kit‐8 (CCK8) assay, apoptosis assay, migration assay, and AM/PI staining. For the CCK8 assay, GL261 cells were seeded in 96 well plated (1 × 10^4^ per well). After treatment with PBS, SAL, PMVS, and PMVS‐P with different concentrations, the cells were treated with 10% CCK8 reagents and incubated at 37 °C for 2 h. Afterward, the absorbance at 450 nm was detected by a microplate reader. For apoptosis assay, GL261 cells were seeded in a 6‐well plated (5 × 10^5^ cells per well). After being treated with PBS, SAL, PMVS, and PMVS‐P (5 µm SAL) for 24 h, cells were digested and detected according to the instructions of the Annexin‐FITC/PI kit (Solarbio, CA1020). To explore the effect of PMVS‐P on the migration ability of tumor cells, we constructed a scratch model and a traswell model in vitro. In the scratch model, GL261 cells were seeded in a 6‐well plate (5 × 10^5^ cells per well). When the cell density reached 80%, a straight line was drawn in the center of the 6‐well plate and subsequently washed with PBS to remove the suspended cells. After being treated with PBS, SAL, PMVS, and PMVS‐P (3 µm SAL) for 24 h, the cell migration rates were tested by microscopic examination. In transwell model, 1 × 10^5^ GL261 cells were seeded in the upper chamber and treated with PBS, SAL, PMVS, and PMVS‐P (3 µm SAL) in solution on both upper and lower chambers for 24 h. Subsequently, cells on the upper side of the transwell membrane were removed, and cells on the lower side were stained with 0.1% crystal violet and observed by optical microscope. For AM/PI staining, GL261 cells were seeded at 5 × 10^5^ per well in confocal dishes and cultured overnight. PBS, SAL, PMVS, and PMVS‐P were added at a concentration of 5 µm. Afterward, the tumor cells were incubated for 48 h and stained by AM/PI according to the instructions of the kit (Solarbio, CA1630).

### Tumor Clone Cluster Formation Assay

GL261 cells were seeded in 6‐well plates at a density of 2 × 10^3^ per well. After overnight culture, tumor cells were treated with PBS, SAL, PMVS, and PMVS‐P (2 µm SAL as the final concentration). After 72 h incubation, the cell supernatant was replaced with a drug‐free complete medium, and incubation continued for 4 days. Afterward, the cell culture supernatant was removed and fixed by adding 4% paraformaldehyde for 15 min. Tumor cells were stained with 0.1% crystal violet for 10 min and washed twice with PBS. Finally, clusters of tumor clones in each group were counted and statistically analyzed.

### GSCs Isolation and Identification

GSCs from the GL261 cell line were isolated by Anti‐Prominin Microbead Kit (Miltenyi, 130‐092‐333). Briefly, 1 × 10^7^ GL261 cells were resuspended in 80 µL PBS, and then 20 µL of anti‐prominin‐1 microbeads were added for 15 min incubation at 4 °C. The mixture was washed twice by adding 1 mL of PBS to remove excess microbeads. Afterward, the mixture was placed in a magnetic field for magnetic sorting. To obtain purified GSCs, two rounds of magnetic sorting were operated. The obtained GSCs were cultured in stem cell medium (DMEM/F12, 20 ng mL^−1^ bFGF, 2% B27, 20 ng mL^−1^ EGF) and 1% hypoxic conditions with ultralow attachment 6 well plate. The markers of GSCs were tested by flow cytometry and CLSM.

### PMVS‐P Uptake and Toxicity in GSCs

Isolated GSCs were seeded in ultralow attachment 6 well plate (1 × 10^5^ cells per well). The GSCs were pretreated with PBS, IgG (10 µg mL^−1^), and anti‐D2DR (10µg mL^−1^) for 2 h. Then, PMV‐NR‐P (NR: 5 µm) was added for 1 h incubation. After being washed 3 times by PBS, GSCs were digested into single cells and the uptake rates of PMV‐NR‐P in GSCs were detected by flow cytometry.

To verify the effect of PDA on cancer stem cell marker expression, different masses of PDA (0, 5, 10, 20, 40, 80 µg) were added and incubated for 48 h. After being digested and fixed with 4% paraformaldehyde, GSCs were stained with FITC‐CD44 and PE‐Sox2 and used for flow cytometry detection. To demonstrate the effect of PMVS‐P on cancer stem cell marker expression, PBS, SAL, PMVS, and PMVS‐P (SAL: 5 µm) were added to GSCs and incubated for 48 h. Afterward, cells were digested, lysed, and boiled by adding a loading buffer. 20 µg proteins per sample were used for the western blotting assay. The proliferation inhibitory effect of PMVS‐P on GSCs was also verified by tumor sphere formation assay. Briefly, GSCs were seeded in ultralow attachment 96 well plate (6 × 10^2^ cells per well). PBS, SAL, PMVS, and PMVS‐P (SAL: 0.5 µm) were added for 5 days of incubation. Afterward, the number and size of the tumor spheres were calculated by optical microscope.

### RNA Isolation and Sequencing

Trizol (1 mL)was added to PBS or PMVS‐P‐treated GL261 cells and lysed for 1 min. Subsequently, cell lysates were collected before adding 0.2 mL of chloroform. Then, the mixture was shaken vigorously for 15 s and left for 3 min. After centrifugation at 10 000 g for 15 min, the upper aqueous phase was taken. Then, the aqueous phase was precipitated with 0.5 mL of isopropanol and centrifuged at 10 000 g for 10 min. RNA precipitates were obtained and washed with 75% ethanol. The obtained pure RNA precipitates were dissolved in RNA‐free water for further use. The RNA purity and integrity were detected by NanoDrop 2000 Spectrophotometer and 2100 Bioanalyzer and 2100 RNA Nano 6000 Assay Kit, respectively. RNA with Poly‐A structure in eukaryotic total RNA was enriched using TIANSeq mRNA Capture Kit (TIANGEN Biotech) from the quality‐assured total RNA. Subsequently, the obtained RNA was used for library construction using the TIANSeq Rapid RNA Library Construction Kit (Illumina platform, TIANGEN Biotech). To construct the transcriptome sequencing library, RNA fragmentation, cDNA one/two strand synthesis, end repair, A‐tail addition, junction ligation, and PCR library enrichment were implemented. Then, the library was diluted to 1 ng µL^−1^. The insert size of the constructed library was detected by Agilent 2100 Bioanalyzer. Q‐PCR was used to quantify the effective concentration of the library and the concentration of the library > 2 nm was considered valid. Afterward, different libraries were pooled according to the effective concentration and the target downstream data volume. PE150 sequencing was performed using Illumina, and 150 bp double‐end sequencing reads were obtained.

### BBB Model In vitro

BBB model was constructed by a 0.4 µm transwell chamber. Briefly, 2.5 × 10^4^ bEnd.3 cells were added to the upper chamber containing mouse type I collagen. After 6 days of incubation, trans‐endothelial electric resistance (TEER) values were measured by Milli cell ERS‐2 (Millipore, USAL). TEER values greater than 180 ΩcM^−^ and had no fluctuations in the liquid level difference for 4 h, which indicates the successful construction of the BBB model. 15 ng mL^−1^ TNF‐α was added for 24 h treatment before different formulations administration. Then, NR, PMV‐NR‐P (NR: 5 µm) were added in the upper chamber for 8 h incubation. To antagonize integrin α6, 100 µg of PMVS‐P was added to 2 µg of integrin antibody and treated for 2 h before adding. Subsequently, NR in the supernatant of the lower chamber was detected and the GSCs in the lower chambers were digested for flow cytometry analysis.

### Animals and GBM Resection Models

All C57BL/6N mice aged 6–8 weeks (18–20 g) were purchased from Charles River (Beijing, China). All animal experiments were performed according to the Guidelines for Care and Use of Laboratory Animals of Chongqing Medical University and National Center for Nanoscience and Technology and were approved by the Animal Ethics Committee of Chongqing Medical University and National Center for Nanoscience and Technology (NCNST21‐2410‐0413). The GBM model was constructed by intracranial injection of Luc‐GL261 cells. Briefly, mice were anesthetized and hairs were removed. A 0.5 cm incision was then cut in the skin of the head and the skull was opened with a drill (1 mm posterior and 2 mm right lateral to the bregma). Microinjector aspirated 3 µL of Luc‐GL261 cells (1.5 × 10^5^ cells) and injected along the cranial foramen (1 mm depth). Sterilize and suture the skin after removing the needle. To obtain a surgical resection model, GBM tissues were resected after 12 days of growth. To obtain a subcutaneous incision model, a 1 cm diameter of skin and subcutaneous tissue was removed by scalpel for further use. The success rate of surgical resection of GBM is ≈80%, and mice with tumor fluorescence intensity < 0.4 × 10^6^ are used as a model for subsequent tumor recurrence experiments.

### Surgical Incision Targeting of PMVS‐P In vivo

The surgical incision targeting ability of PMVS‐P was tested by In Vivo Imaging Systems (IVIS). Briefly, DiR and PMV‐DiR‐P (DiR: 40 µg) were injected from the tail vein in the subcutaneous surgical incision mouse model and in the orthotopic GBM surgical incision mouse model. Subsequently, we monitored the aggregation of DiR at the surgical incision site at 3, 6, 12, 24, 48, and 72 h. To detect the aggregation of nanoparticles in the brain and major organs, we dissected the mice 48 h after injection and removed the brain, heart, liver, spleen, lung, and kidneys to observe the aggregation of DiR in vital organs by IVIS.

### Therapeutic Efficacy and Survival Experiments

GBM‐bearing mice were treated with surgical resection on day 12. Subsequently, mice were randomly divided into four groups and injected with PBS, SAL, PMVS, and PMVS‐P (SAL: 100 µg per mouse) every other day. The signal intensity of Luc‐GL261 was detected and recorded by IVIS. For survival experiments, treatments were the same as previously described, and the death rate and body weight of the mice were recorded every day.

### Biosafety Assessment In vivo

To evaluate the toxicity of PMVS‐P, major organs (heart, liver, spleen, lung, and kidneys) of treated mice were dissected and fixed with 4% paraformaldehyde for 1 day, followed by sectioning and H&E staining. Mouse blood after treatment was centrifuged at 800 g for 30 min and the supernatant was subsequently used for the determination of aspartate transaminase (AST), glutamate transaminase (ALT), alkaline phosphatase (ALP), glucose (Glu), blood urea nitrogen (BUN) and cholesterol (CHO1).

### Statistical Analysis

Data were shown as the mean ± standard deviation (S.D.). The significance of the data was determined by the Student's *t*‐test performed by Graph Pad Prism8 software. *p*‐values < 0.05 were regarded as statistically significant. *p < 0.05; **p < 0.01; ***p < 0.001; ****p < 0.0001.

## Conflict of Interest

The authors declare that they have no conflict of interest.

## Supporting information



Supporting Information

## Data Availability

The data that support the findings of this study are available from the corresponding author upon reasonable request.

## References

[1] L. R. Schaff , I. K. Mellinghoff , JAMA, J. Am. Med. Assoc.JAMA, J. Am. Med. Assoc. 2023, 329, 574. 10.1001/jama.2023.0023 PMC11445779 36809318

[2] a) A. Desjardins , M. Gromeier , J. E. Herndon , N. Beaubier , D. P. Bolognesi , A. H. Friedman , H. S. Friedman , F. McSherry , A. M. Muscat , S. Nair , K. B. Peters , D. Randazzo , J. H. Sampson , G. Vlahovic , W. T. Harrison , R. E. McLendon , D. Ashley , D. D. Bigner , New Engl J Med 2018, 379, 150; 29943666 10.1056/NEJMoa1716435 PMC6065102

[3] C. Chen , W. Q. Jing , Y. Chen , G. Y. Wang , M. Abdalla , L. Gao , M. S. Han , C. D. Shi , A. N. Li , P. Sun , X. Jiang , Z. M. Yang , S. C. Zhang , J. Zhang , C. W. Tang , Y. Liu , R. Zhang , F. B. Xu , B. X. Dong , X. E. Li , M. L. Liu , B. M. Qiang , Y. H. Sun , X. Wei , J. Li , Q. Y. Hu , X. Y. Jiang , Sci. Transl. Med. 2022, 14, eabn1128. 10.1126/scitranslmed.abn1128 35921473

[4] a) B. Z. Li , X. P. Zhang , Z. L. Wu , T. J. Chu , Z. L. Yang , S. Xu , S. Y. Wu , Y. K. Qie , Z. F. Lu , F. L. Qi , M. G. Hu , G. D. Zhao , J. Y. Wei , Y. L. Zhao , G. J. Nie , H. Meng , R. Liu , S. P. Li , Adv. Sci. 2022, 9, e2200477; 10.1002/advs.202200477 PMC9284141 35524631

[5] B. Z. Li , T. J. Chu , J. Y. Wei , Y. L. Zhang , F. L. Qi , Z. F. Lu , C. Gao , T. J. Zhang , E. S. Jiang , J. C. Xu , J. Q. Xu , S. P. Li , G. J. Nie , Nano Lett. 2021, 21, 2588. 33650872 10.1021/acs.nanolett.1c00168

[6] M. X. Li , Y. Liu , J. P. Chen , T. T. Liu , Z. X. Gu , J. Q. Zhang , X. C. Gu , G. J. Teng , F. Yang , N. Gu , Theranostics 2018, 8, 4870. 30429874 10.7150/thno.27466 PMC6217069

[7] S. Wang , T. Che , A. Levit , B. K. Shoichet , D. Wacker , B. L. Roth , NatureNature 2018, 555, 269. 10.1038/nature25758 PMC5843546 29466326

[8] a) S. P. Caragher , J. M. Shireman , M. Huang , J. Miska , F. Atashi , S. Baisiwala , C. H. Park , M. R. Saathoff , L. Warnke , T. Xiao , M. S. Lesniak , C. D. James , H. Meltzer , A. K. Tryba , A. U. Ahmed , J. Neurosci. 2019, 39, 1982. 30651332 10.1523/JNEUROSCI.1589-18.2018 PMC6507082

[9] a) D. Wu , J. Zhou , Y. R. Zheng , Y. Y. Zheng , Q. Zhang , Z. C. Zhou , X. J. Chen , Q. Chen , Y. P. Ruan , Y. Wang , Z. Chen , Nat. Commun. 2023, 14, 7147; 37932306 10.1038/s41467-023-43070-z PMC10628287

[10] Y. Liu , C. K. K. Choi , H. Hong , Y. Xiao , M. L. Kwok , H. Liu , X. Y. Tian , C. H. J. Choi , ACS Nano 2021, 15, 13871. 34379407 10.1021/acsnano.1c06081

[11] a) A. Shariati , T. Ebrahimi , P. Babadinia , F. S. Shariati , R. A. Cohan , Sci Rep‐Uk 2023, 13, 4520; 10.1038/s41598-023-31252-0 PMC10024681 36934115

[12] C. Pan , J. J. Li , W. L. Hou , S. S. Lin , L. Wang , Y. Pang , Y. F. Wang , J. Y. Liu , Adv. Mater. 2021, 33, 2007379. 10.1002/adma.202007379 33629757

[13] J. Kim , Y. Zhu , S. H. Chen , D. D. Wang , S. Y. Zhang , J. X. Xia , S. Y. Li , Q. J. Qiu , H. Lee , J. X. Wang , J. Nanobiotechnol. 2023, 21, 253. 10.1186/s12951-023-02006-x PMC10401762 37542285

[14] S. N. Yu , S. H. Kim , K. Y. Kim , J. H. Ji , Y. K. Seo , H. S. Yu , S. C. Ahn , Oncol Rep 2017, 37, 3321. 28498472 10.3892/or.2017.5615

[15] B. J. Chen , X. R. Wu , Y. S. Ruan , Y. L. Zhang , Q. C. Cai , L. Zapata , C. I. Wu , P. Lan , H. J. Wen , Natl. Sci. Rev. 2022, 9, nwac250. 36694802 10.1093/nsr/nwac250 PMC9869076

[16] J. C. Chang , Medicine 2016, 95, S20. 27611935 10.1097/MD.0000000000004766 PMC5599212

[17] J. F. Zhou , S. L. Liu , Y. Wang , W. Dai , H. L. Zou , S. B. Wang , J. Zhang , J. X. Pan , Mol Cancer 2021, 20, 47. 33658013 10.1186/s12943-021-01334-6 PMC7927384

[18] J. Park , T. F. Brust , H. J. Lee , S. C. Lee , V. J. Watts , Y. Yeo , ACS Nano 2014, 8, 3347. 24628245 10.1021/nn405809c PMC4107448

[19] Q. You , F. M. Liang , G. G. Wu , F. F. Cao , J. Y. Liu , Z. H. He , C. Wang , L. Zhu , X. Y. Chen , Y. L. Yang , Adv. Mater. 2024, 36, 2306583. 10.1002/adma.202306583 37713652

[20] a) H. Kim , K. Leng , J. Park , A. G. Sorets , S. Kim , A. Shostak , R. J. Embalabala , K. Mlouk , K. A. Katdare , I. V. L. Rose , S. M. Sturgeon , E. H. Neal , Y. Ao , S. N. Wang , M. V. Sofroniew , J. M. Brunger , D. G. McMahon , M. S. Schrag , M. Kampmann , E. S. Lippmann , Nat. Commun. 2022, 13, 6581; 36323693 10.1038/s41467-022-34412-4 PMC9630454

[21] Z. H. Peng , X. C. Zhang , L. Yuan , T. Li , Y. J. Chen , H. Tian , D. D. Ma , J. Deng , X. W. Qi , X. T. Yin , J. Nanobiotechnol. 2021, 19, 383. 10.1186/s12951-021-01130-w PMC8607565 34809612

[22] S. Liu , T. S. Zhang , S. S. Li , Q. Y. Wu , K. X. Wang , X. C. Xu , M. Z. Lu , R. G. Shao , W. L. Zhao , H. Y. Liu , Small 2023, 19, e2206503. 36587973 10.1002/smll.202206503

[23] S. Y. Zhang , M. Zhong , H. B. Zhu , Q. H. You , H. Yuan , Y. P. Li , Cancer Genet‐Ny 2023, 278, 71. 10.1016/j.cancergen.2023.09.001 37729778

[24] M. C. Tung , Y. W. Lin , W. J. Lee , Y. C. Wen , Y. C. Liu , J. Q. Chen , M. Hsiao , Y. C. Yang , M. H. Chien , Cell Death Dis. 2022, 13, 400. 35461314 10.1038/s41419-022-04828-3 PMC9035181

[25] K. Pal , T. Hussain , H. Xie , S. D. Li , P. Yang , A. Mansfield , Y. Y. Lou , S. Chowdhury , D. Mukhopadhyay , Front. Oncol. 2022, 12, 959500. 36072788 10.3389/fonc.2022.959500 PMC9441878

[26] F. Gemignani , S. Landi , V. Moreno , L. Gioia‐Patricola , A. Chabrier , E. Guino , M. Navarro , M. Cambray , G. Capellà , F. Canzian , B. C. Cancer , Cancer Epidem Biomar 2005, 14, 1633. 10.1158/1055-9965.EPI-05-0057 16030094

