# Supplementary material for: Neurotransmitter‐Mimicking Nanovesicles Facilitate Postoperative Glioblastoma Stem Cell‐Specific Treatment for Preventing Tumor Recurrence
Source: Adv Sci (Weinh). 2024 Dec 25;12(7):2409713. doi: 10.1002/advs.202409713 (PMC11831431; doi:10.1002/advs.202409713)
Supplement: Supplementary file 1 — Supporting Information [file ADVS-12-2409713-s001.docx]

Supplementary Materials for

**Neurotransmitter-mimicking nanovesicles-mediated postoperative glioblastoma stem cell–specific treatment for preventing tumor recurrence**

Fuming Liang ^1, 2, 3, #^, Qing You ^2, 5, #^, Bin Yu ^4^, Chen Wang ^2, 3^, Yanlian Yang ^2, 3, *^, Ling Zhu ^2, 3, *^, Zhaohui He ^1, *^

^1^ Department of Neurosurgery, The First Affiliated Hospital of Chongqing Medical University, 1 Friendship Road, Chongqing 400016, P. R. China

^2^ CAS Key Laboratory of Standardization and Measurement for Nanotechnology, CAS Key Laboratory for Biomedical Effects of Nanomaterials and Nanosafety, CAS Center for Excellence in Nanoscience, National Center for Nanoscience and Technology, Beijing 100190, P. R. China

^3^ University of Chinese Academy of Sciences, Beijing 100049, P. R. China

^4^ Department of Radiology, The First Affiliated Hospital of Chongqing Medical University, 1 Friendship Road, Chongqing 400016, P. R. China

^5^ Department of Diagnostic Radiology, Yong Loo Lin School of Medicine, National University of Singapore, Singapore 119074, Singapore

^*^ Corresponding author

^#^ These authors contributed equally to this work

Email: geno_he@163.com, zhul@nanoctr.cn, yangyl@nanoctr.cn

**
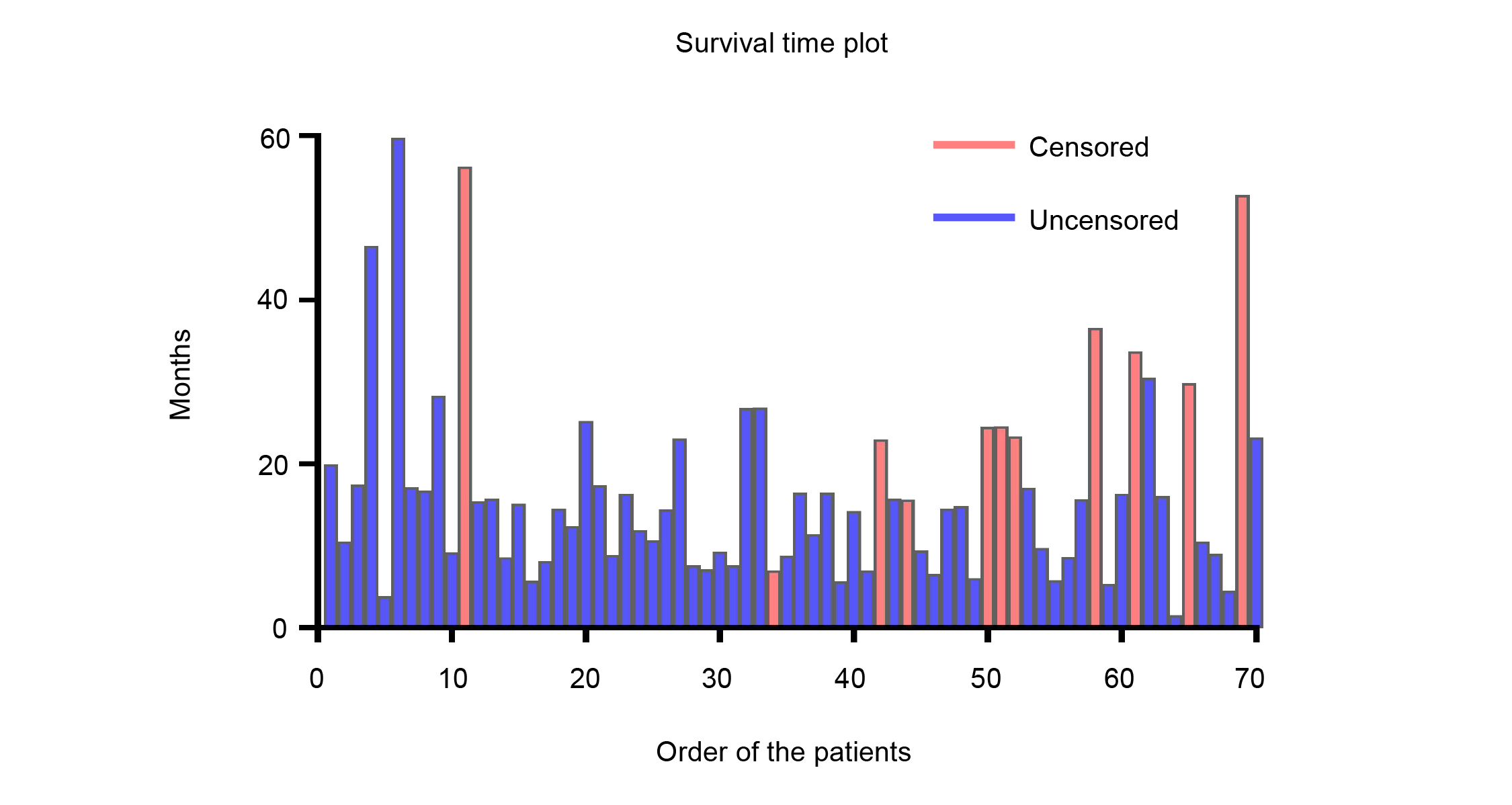
**

**Figure S1.** Distribution of patients with right-censored data in Sox2 and CD44 gene-related survival analysis.

**
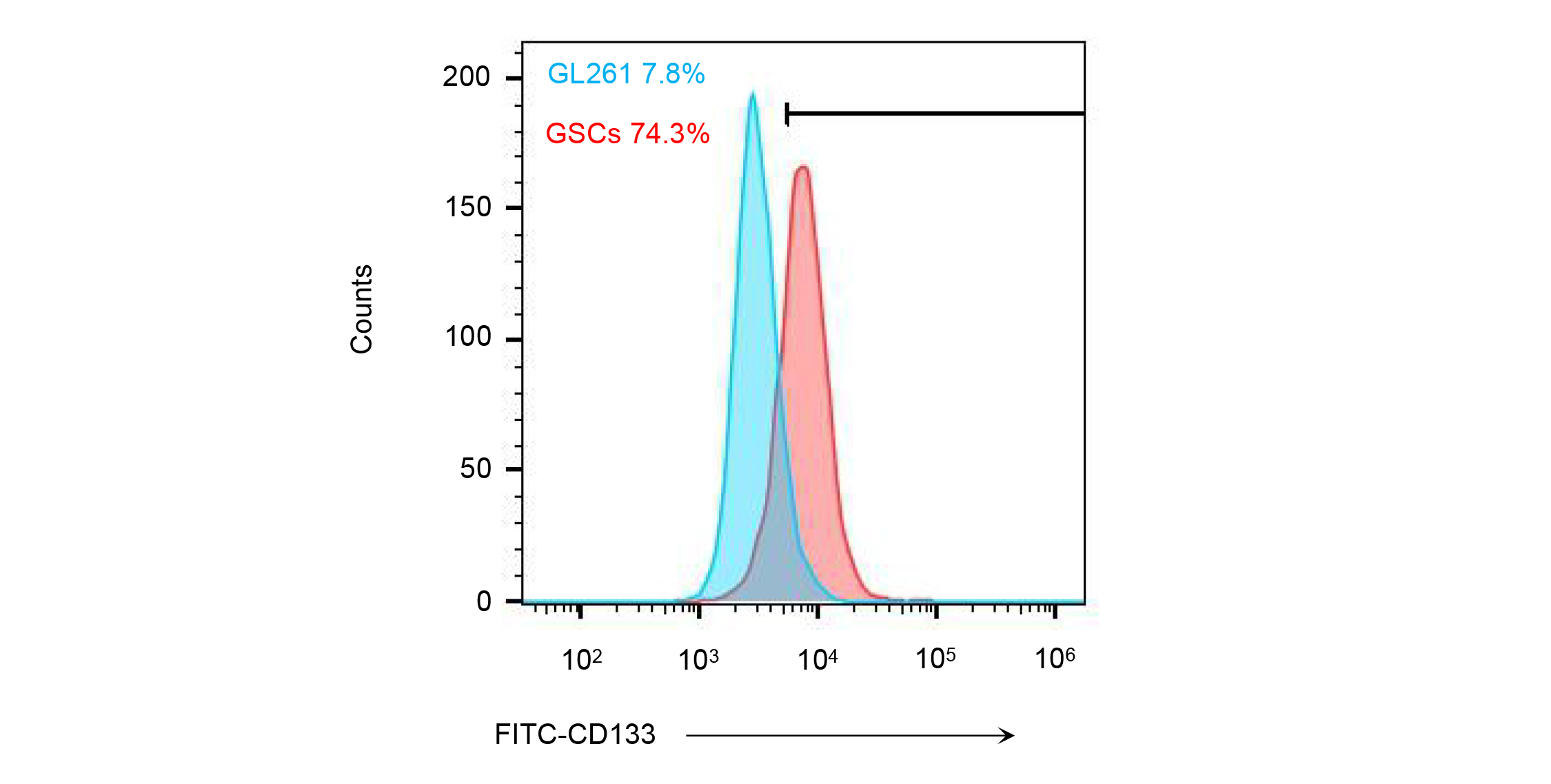
**

**Figure S2.** Expression of CD133 in GL261 and sorted GSCs tested by flow cytometry.


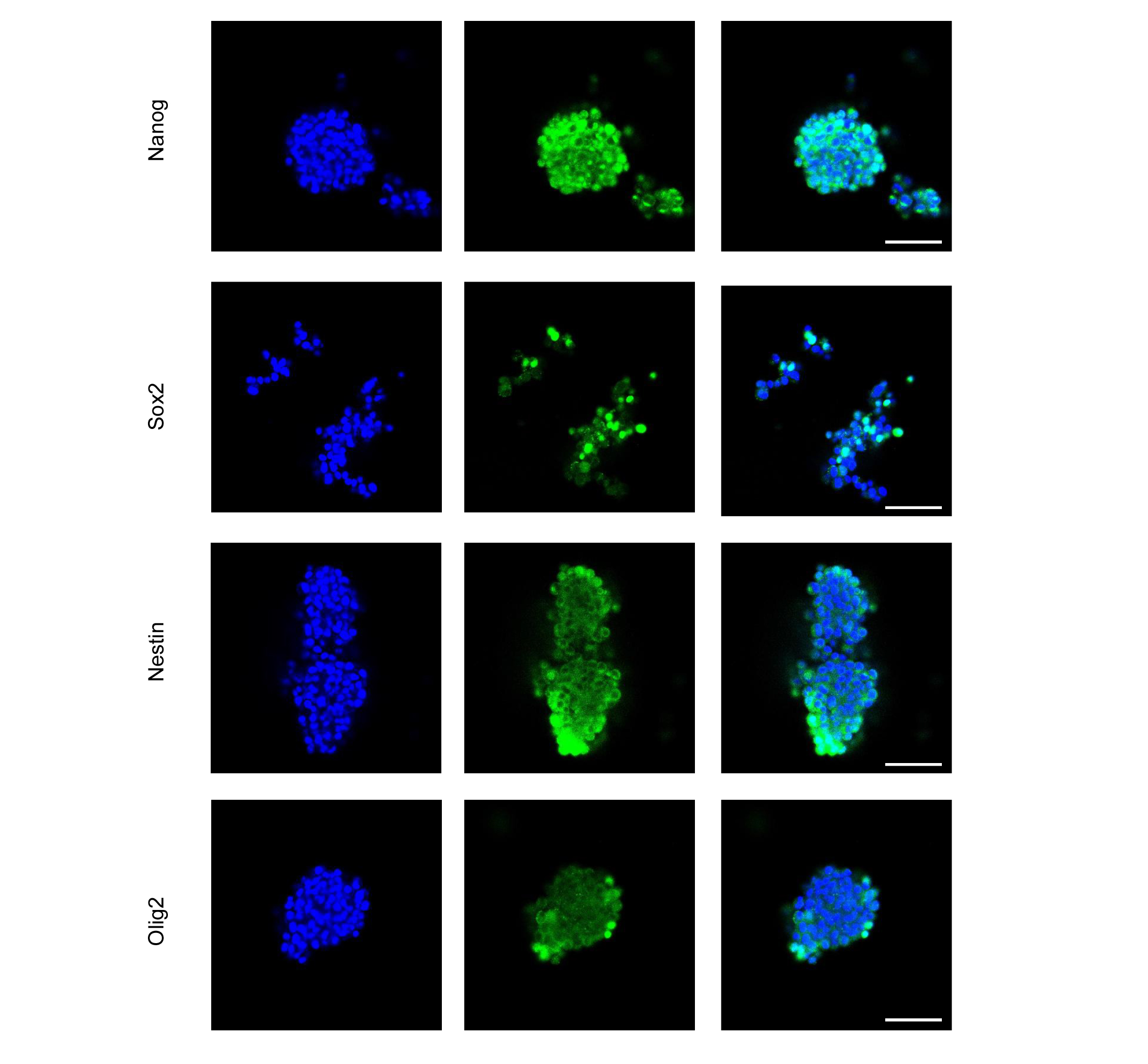


**Figure S3.** The expression of Nanog, Sox2, Nestin and Olig2 in GSC spheres after magnetic sorting and 5 days incubation tested by confocal laser scanning microscope (CLSM). DAPI: blue; Nanog, Sox2, Nestin and Olig2: green. Scale bar: 100 μm.

**
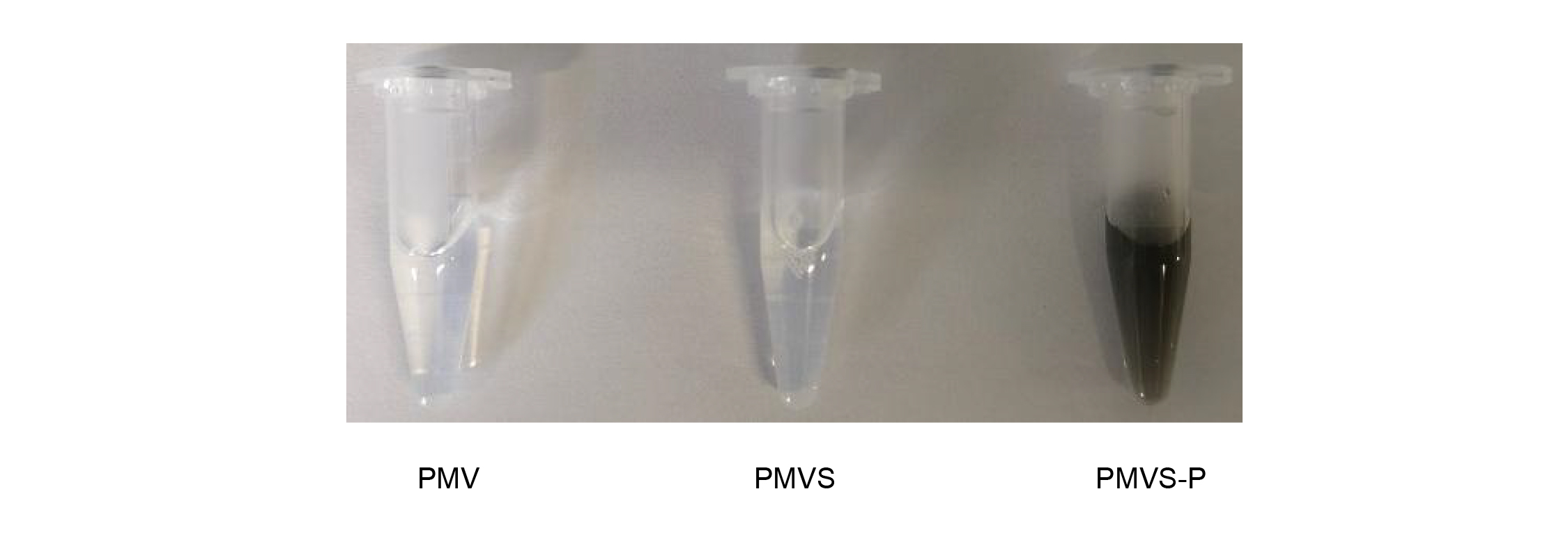
**

**Figure S4.** Optical images of platelet membrane vesicle (PMV), platelet membrane vesicle loaded with salinomycin (PMVS), and platelet membrane vesicle loaded with salinomycin and modification with polydopamine (PMVS-P).


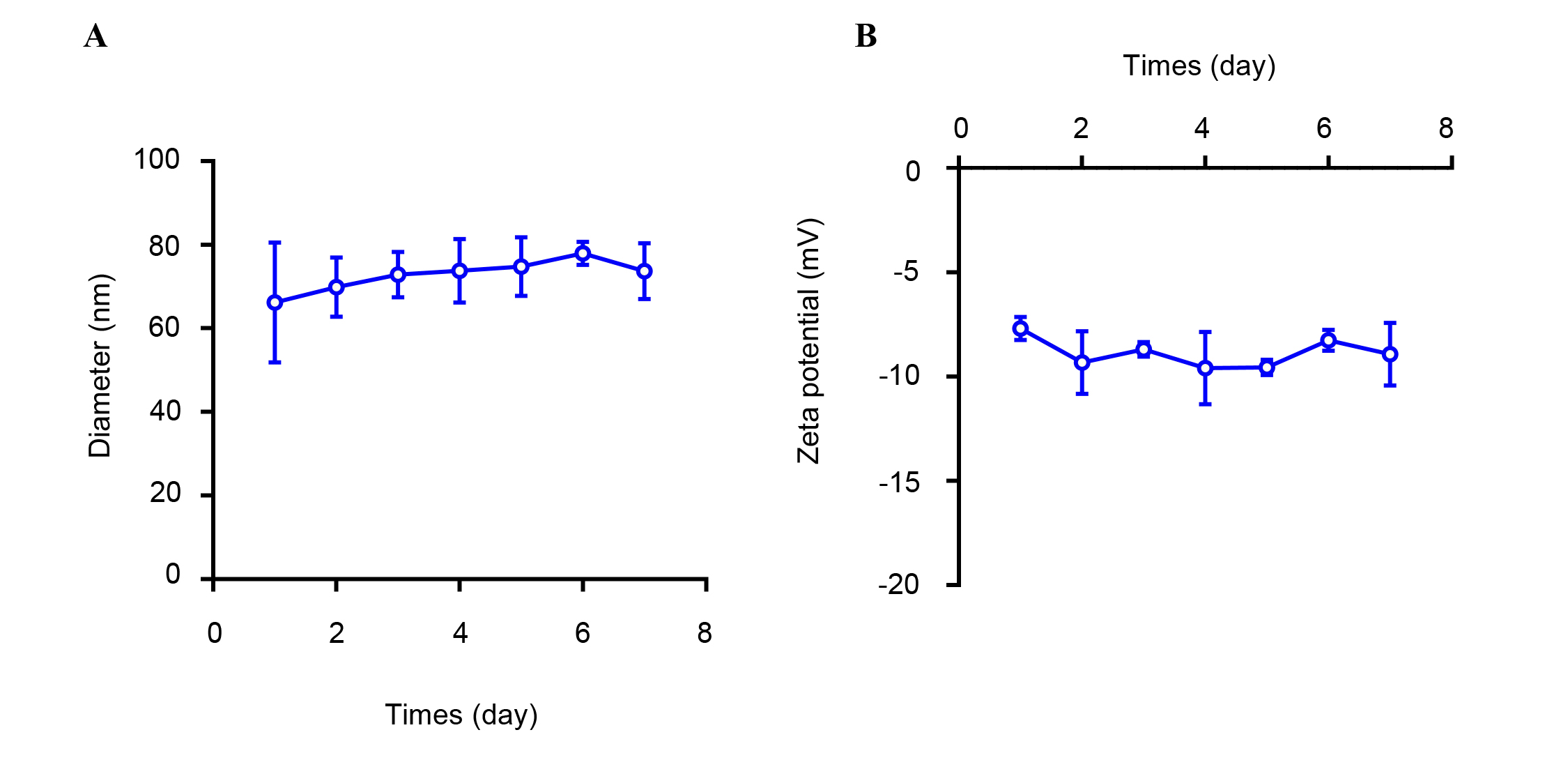


**Figure S5.** DLS long-term detection for PMVS-P. (A) PMVS-P continuous one-week particle size monitoring in DLS testing. (B) PMVS-P continuous one-week zeta potential monitoring in DLS testing.


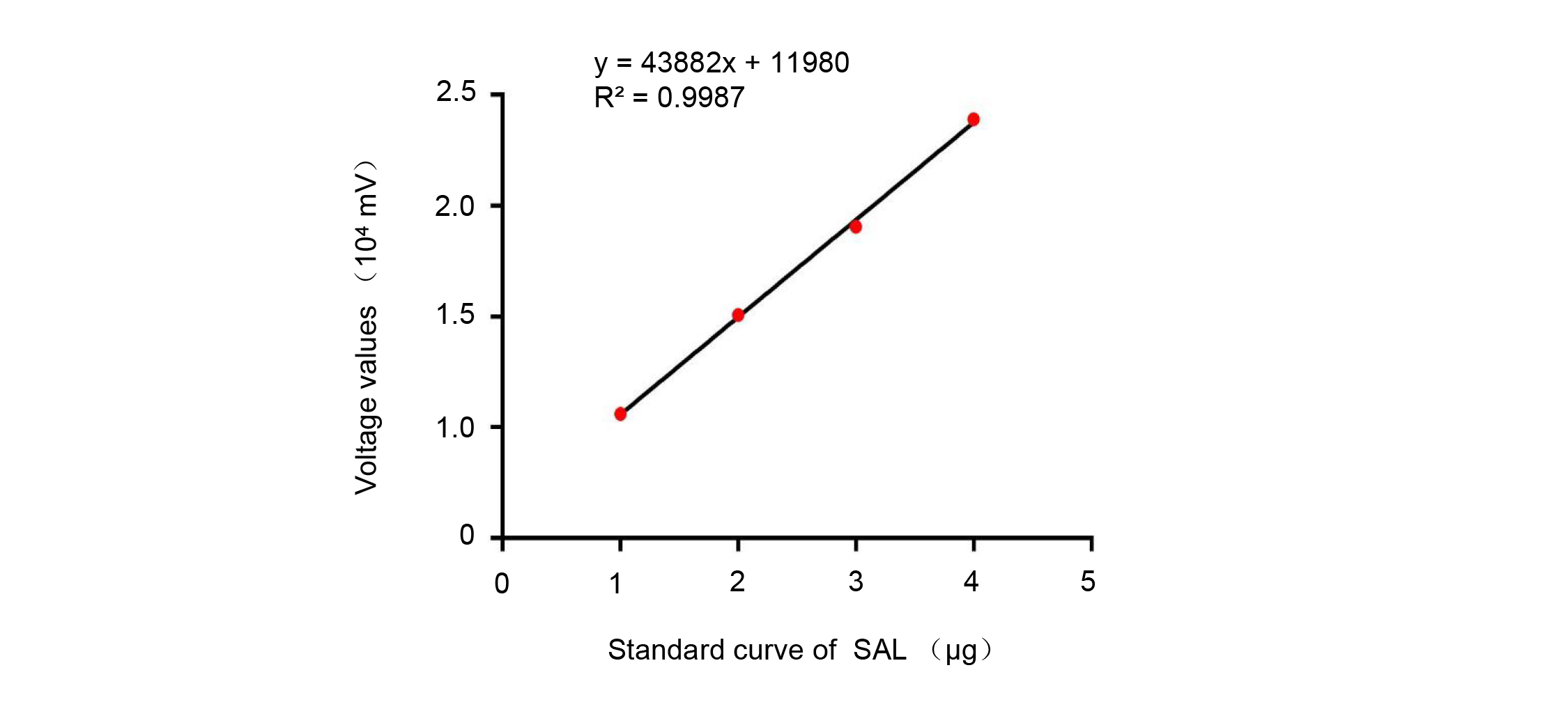


**Figure S6.** Standard curve of salinomycin (SAL) in high performance liquid chromatography (HPLC). The curves represent the voltage values corresponding to different masses of SAL (1 μg, 2 μg, 3 μg, 4 μg) in HPLC.


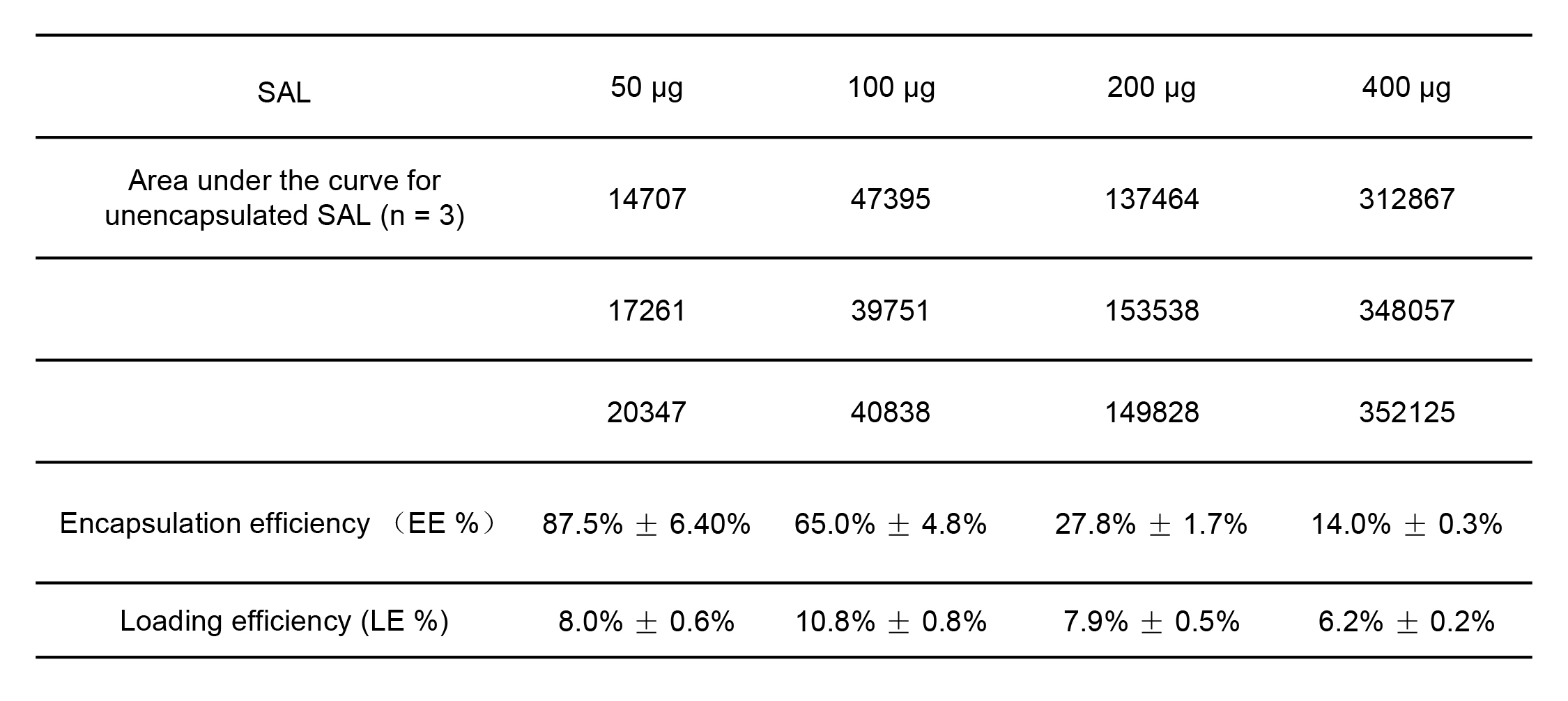


**Figure S7.** Area under the curve of the remaining SAL in the supernatant after synthesizing PMVS-P for different masses of SAL tested by HPLC.


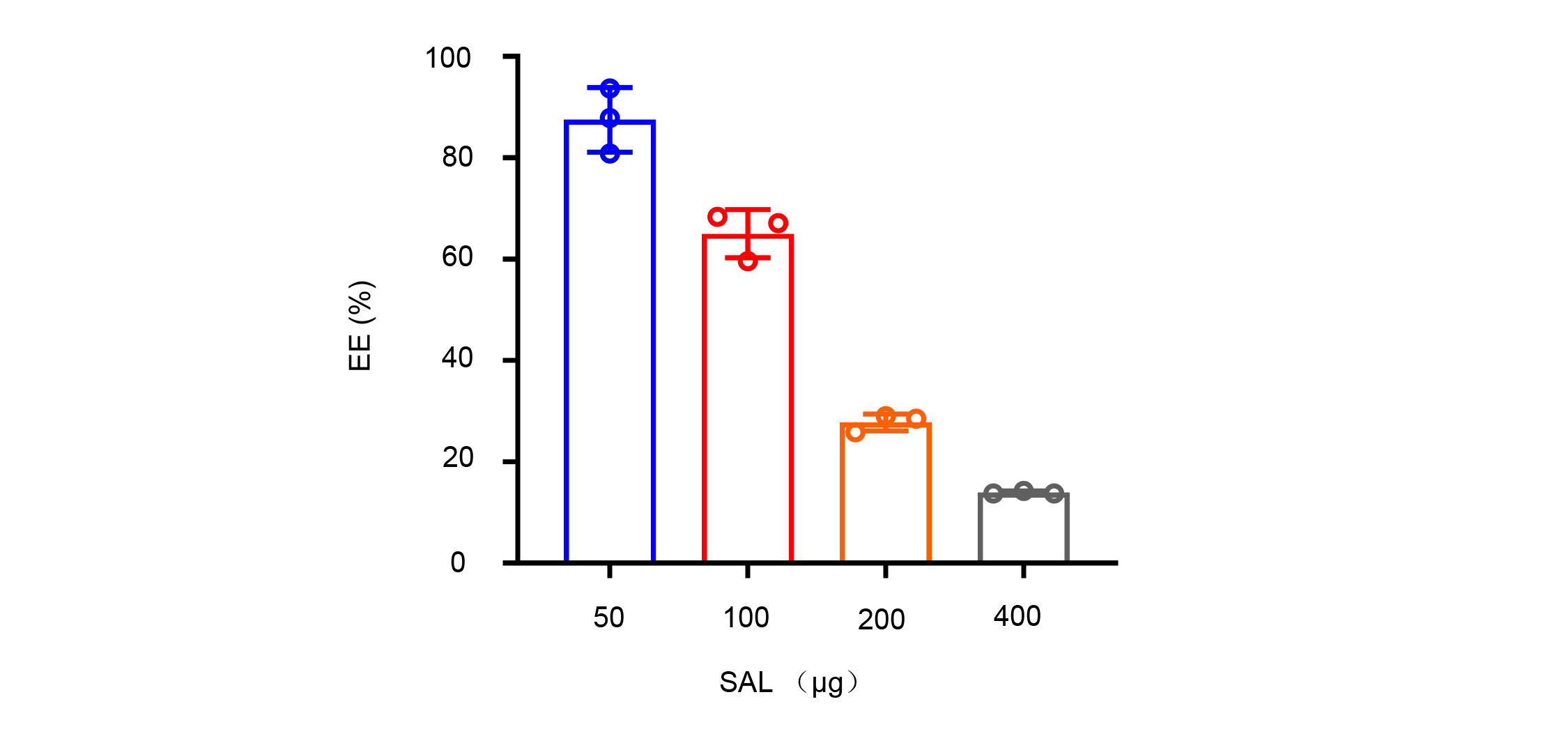


**Figure S8.** Encapsulation efficiency of different amounts of SAL in PMVS-P tested by HPLC.


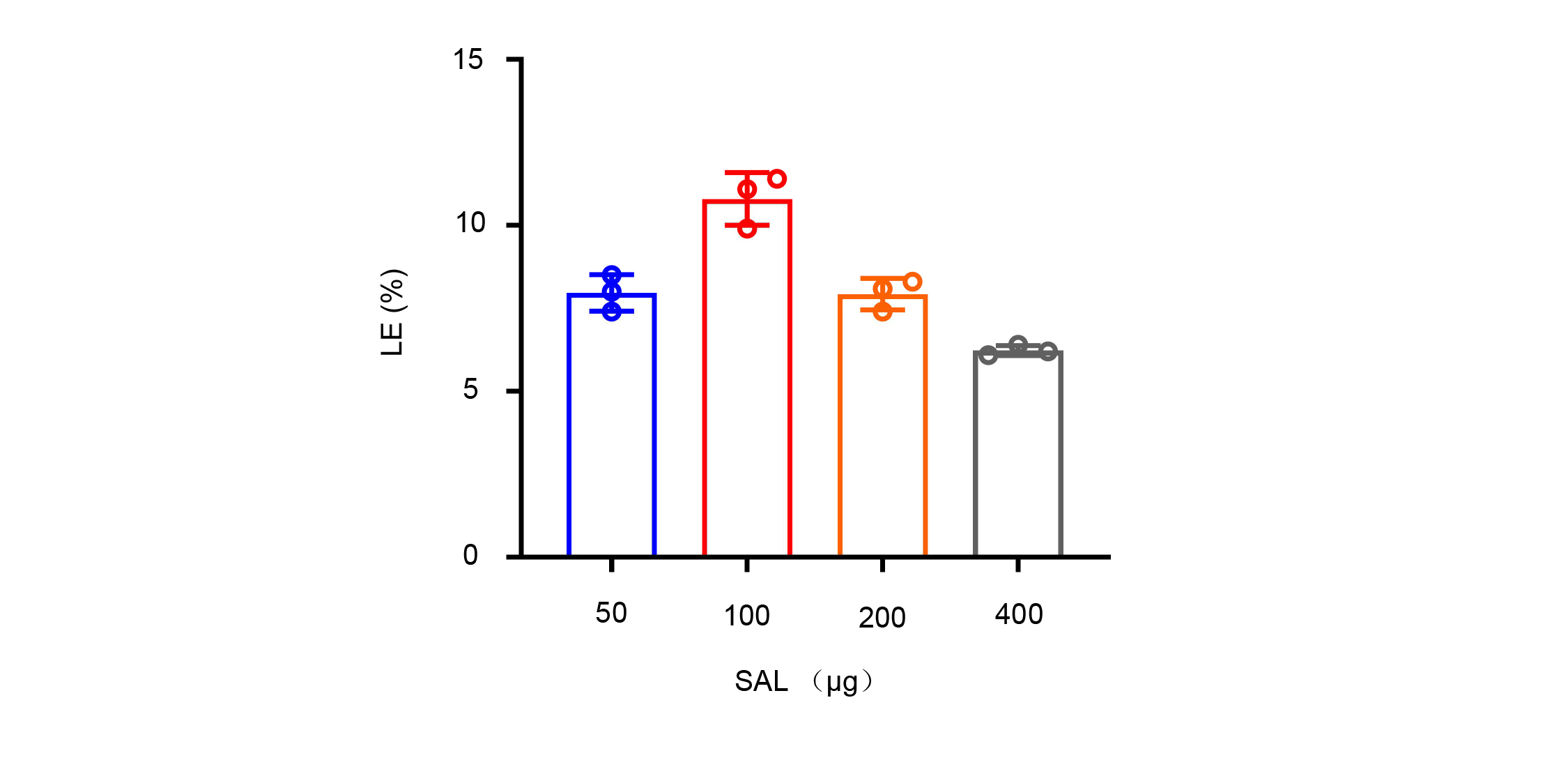


**Figure S9.** Loading efficiency of different amounts of SAL in PMVS-P tested by HPLC.


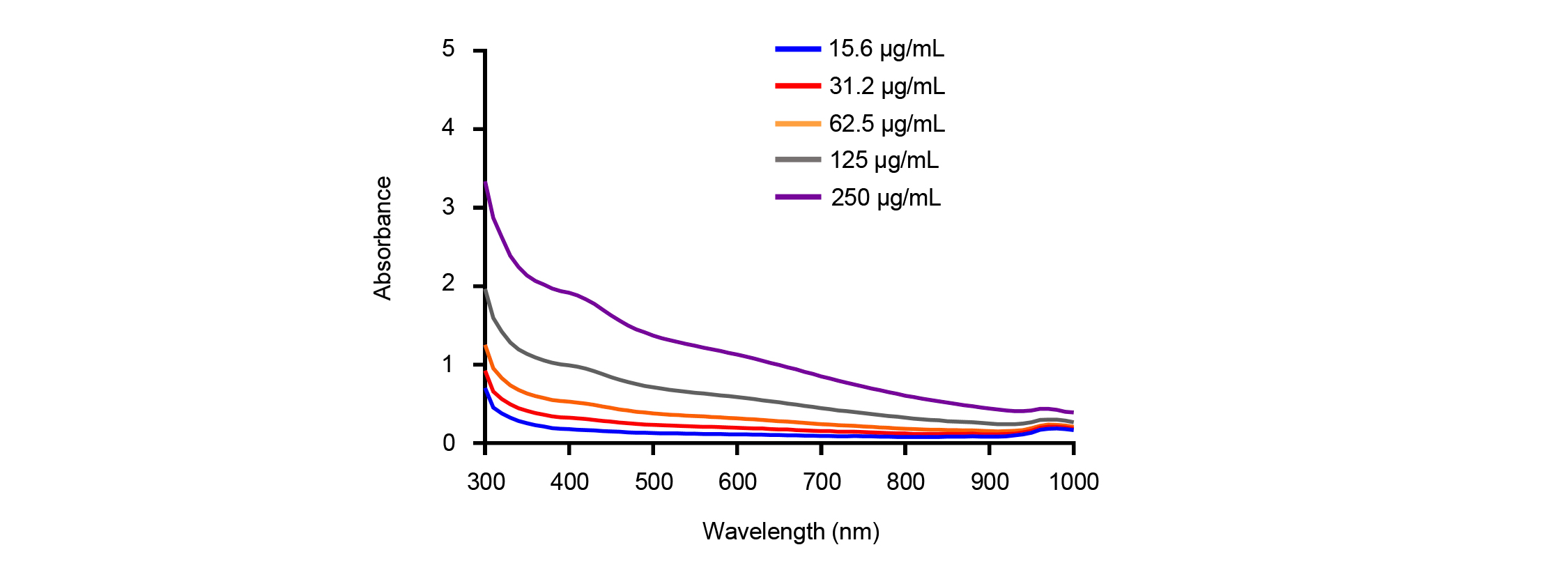


**Figure S10.** Ultraviolet absorption spectra of different concentrations (15.6, 31.2, 62.5, 125, 250 μg/mL) of polydopamine.


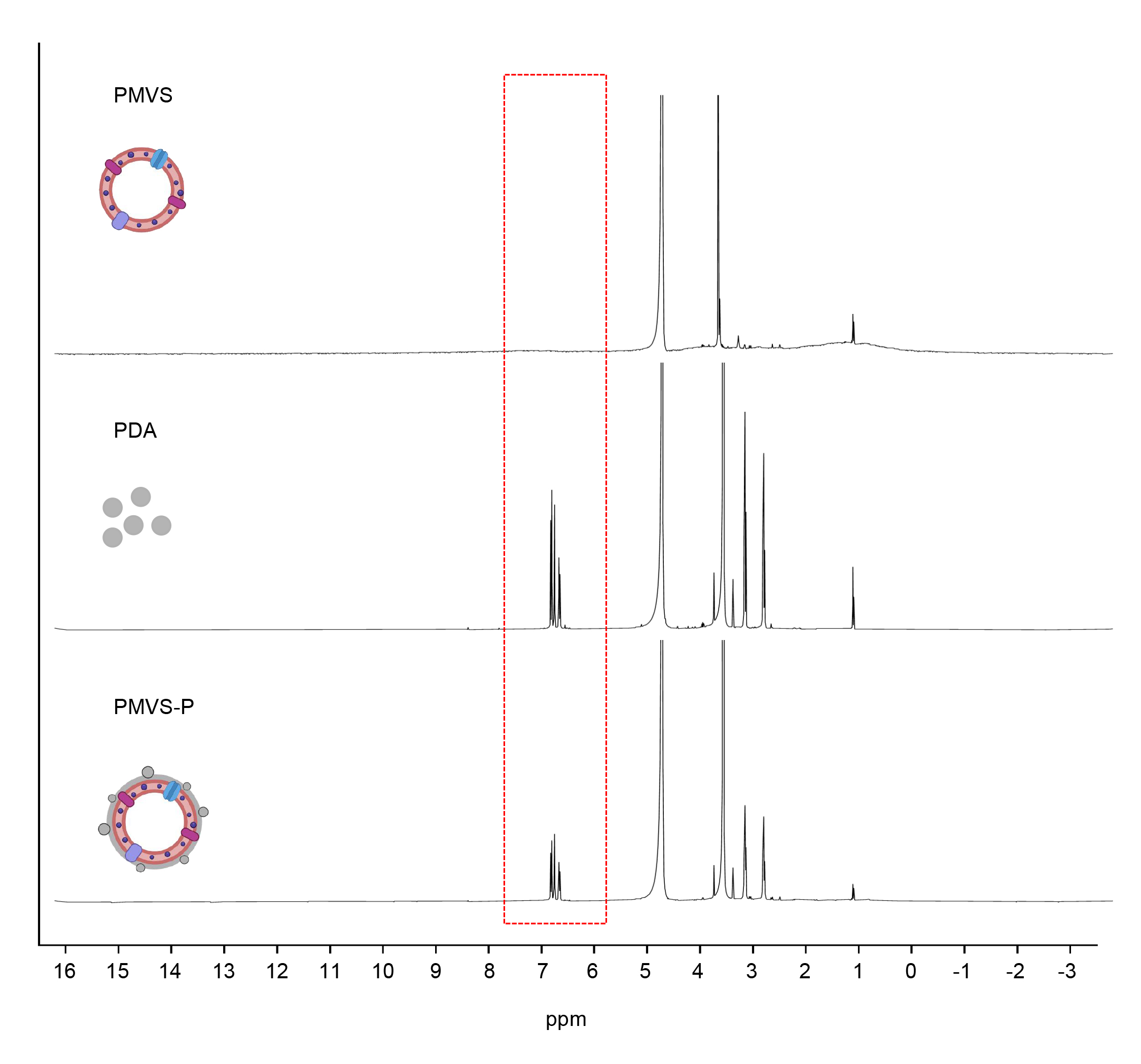


**Figure S11.** (1)H-NMR detection spectra of PMVS, PDA and PMVS-P nanomaterials. The absorption peak corresponding to the benzene ring is marked by a red box.


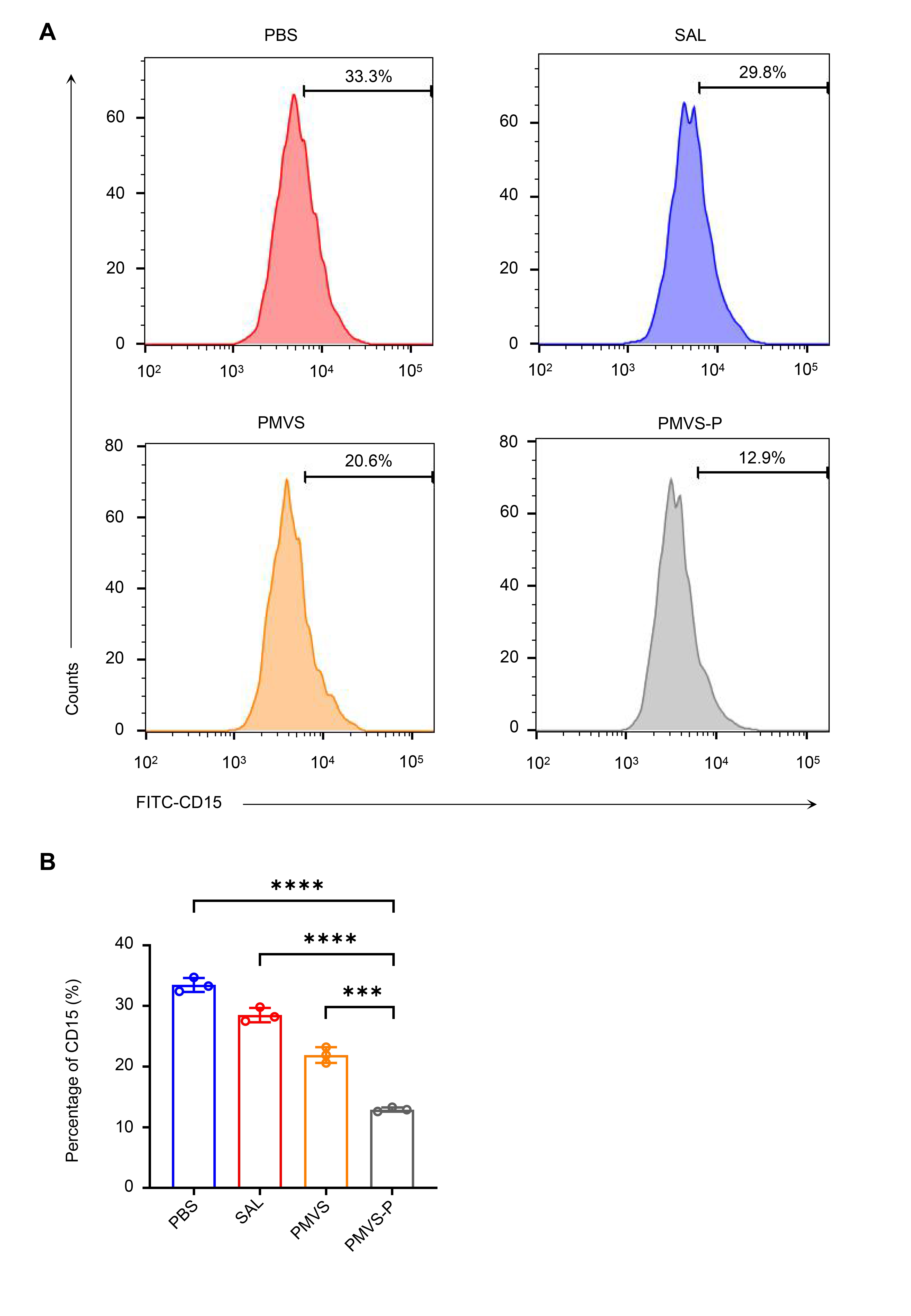


**Figure S12.** Flow cytometry (A) and the relevant quantitative analysis (B) of CD15 expression in GBM cells after PBS, SAL, PMVS, and PMVS-P treatment.


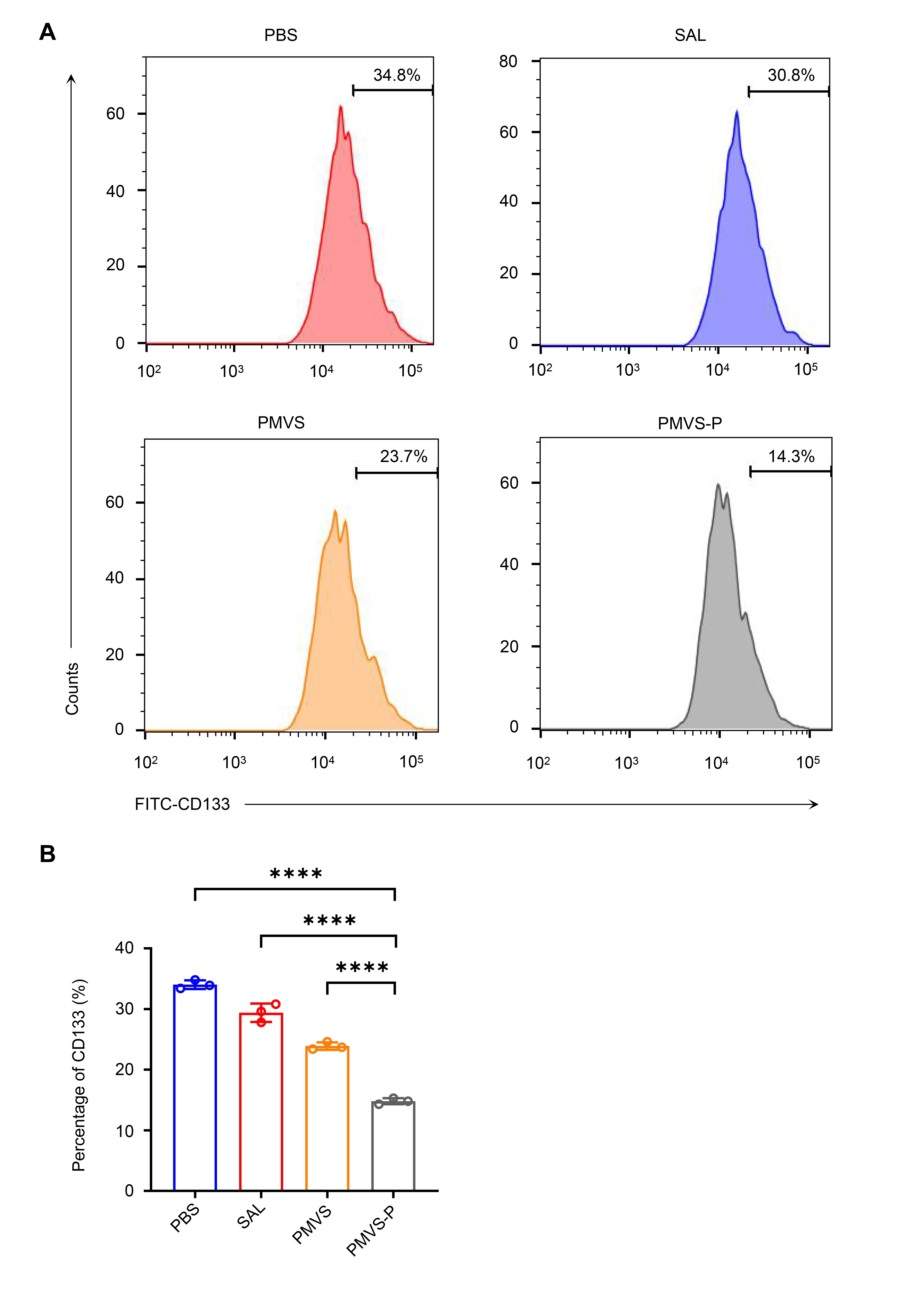


**Figure S13.** Flow cytometry (A) and the relevant quantitative analysis (B) of CD133 expression in GBM cells after PBS, SAL, PMVS, and PMVS-P.

**
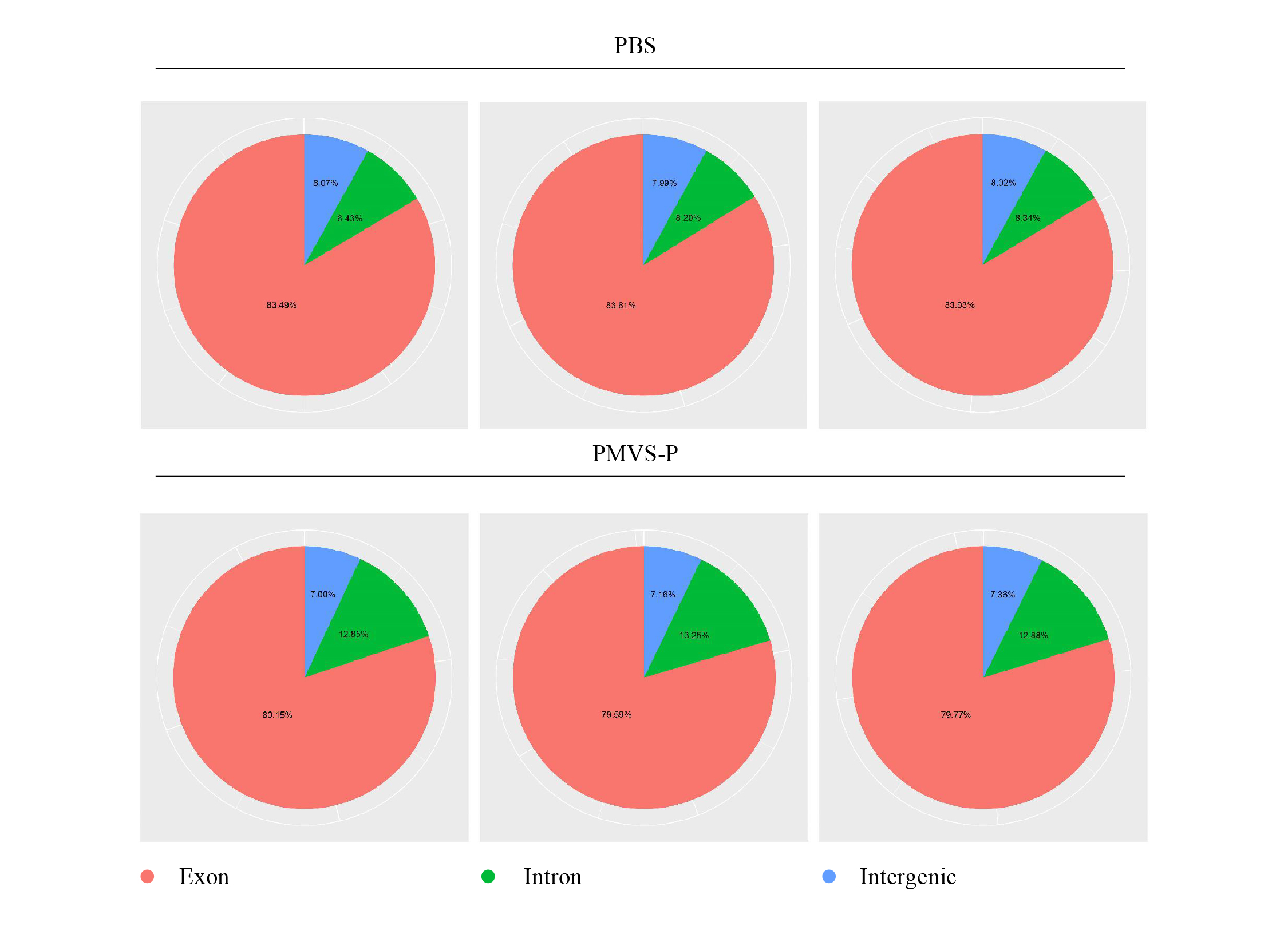
**

**Figure S14.** Reads mapped to genome regions in PBS and PMVS-P groups.


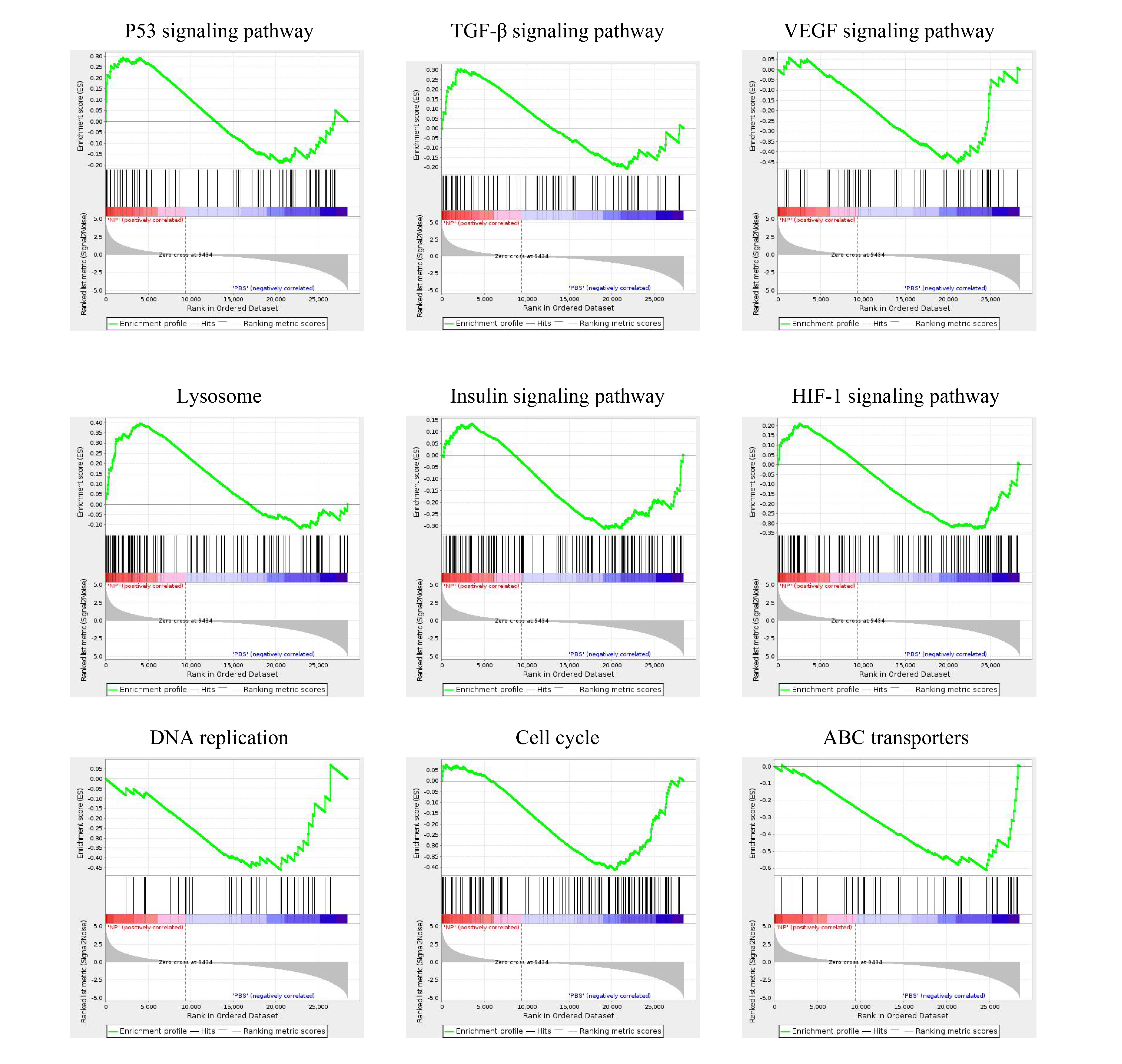


**Figure S15.** GSEA plots showing the cellular component, signaling pathway and molecular fucntion of DEGs altered by PMVS-P in GBM cells. NP: PMVS-P nanoparticle.


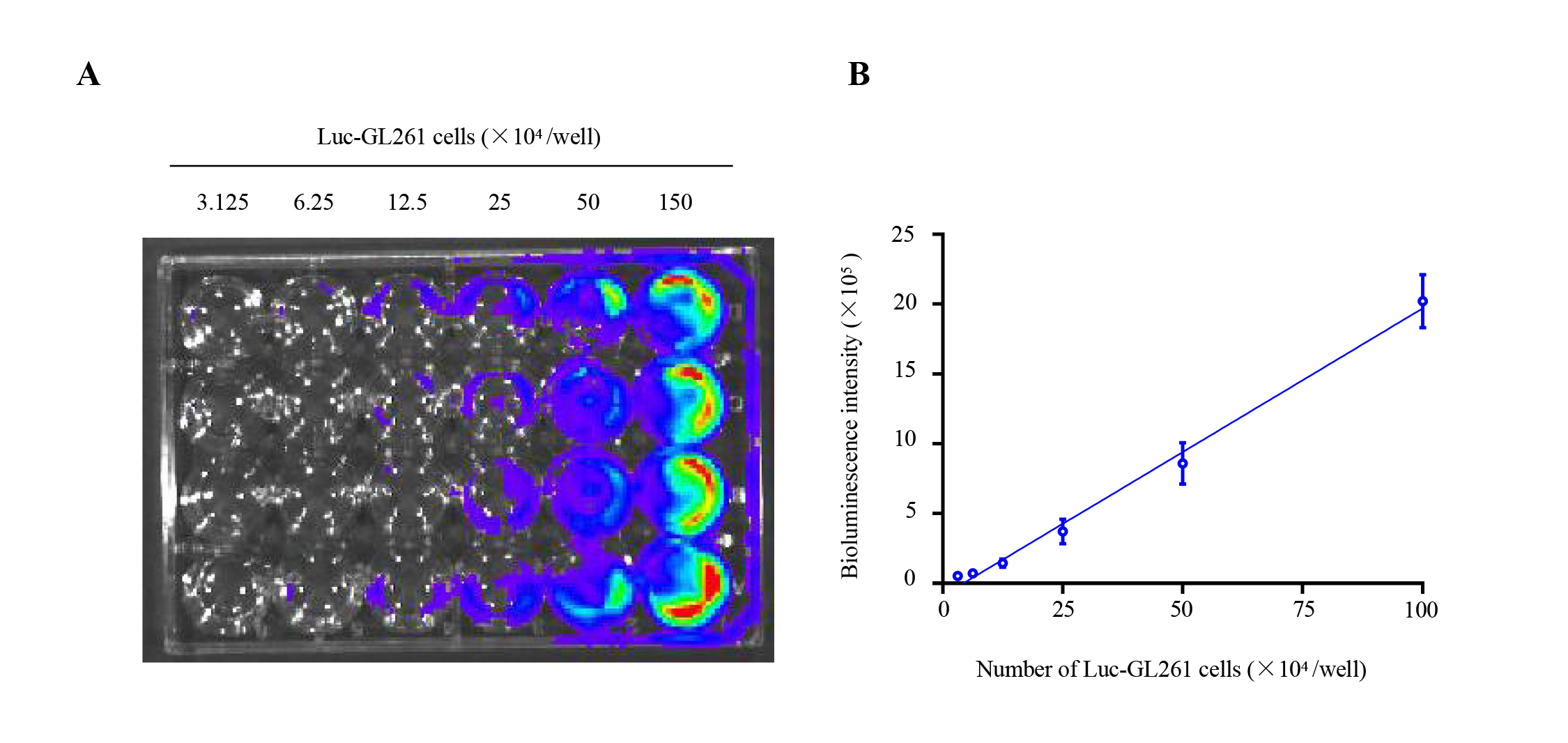


**Figure S16.** Fluorescence intensity corresponding to different numbers of Luc-GL261 tumor cells (*n* = 4).


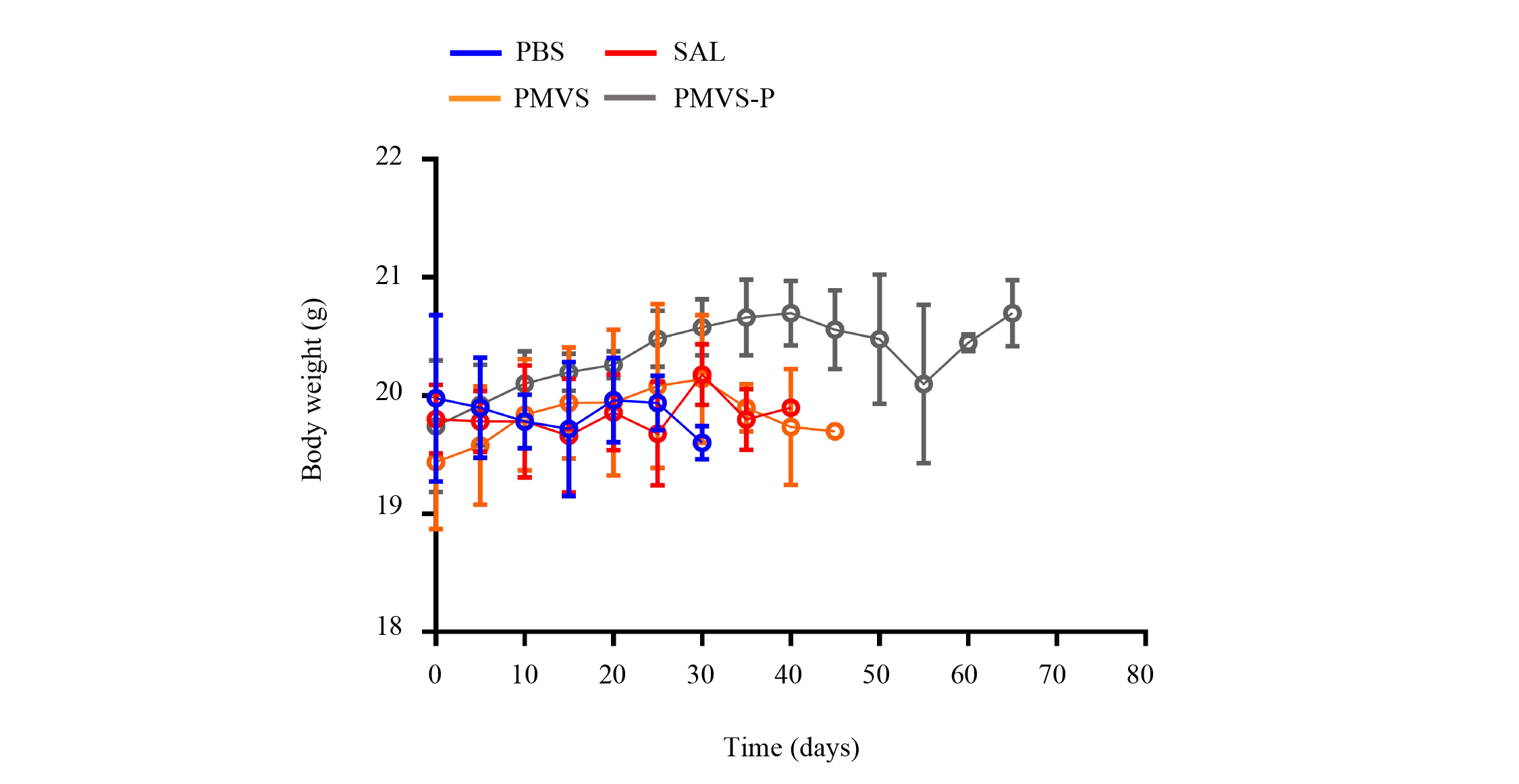


**Figure S17.** Body weight curves of tumor-bearing mice after PBS, SAL, PMVS and PMVS-P treatment.

|  | Days | 13 d (resection) | 17 d | 21 d | 25 d |
| --- | --- | --- | --- | --- | --- |
| PBS  (×10^6^) | Mean | 0.25 | 2.67 | 3.57 | 6.43 |
|  | Variance | 0.01 | 1.82 | 1.17 | 6.35 |
|  | Standard deviation | 0.11 | 1.35 | 1.08 | 2.52 |
| SAL  (×10^6^) | Mean | 0.22 | 1.90 | 2.17 | 3.93 |
|  | Variance | 0.03 | 0.61 | 0.16 | 1.00 |
|  | Standard deviation | 0.17 | 0.78 | 0.40 | 1.00 |
| PMVS  (×10^6^) | Mean | 0.18 | 1.70 | 1.52 | 2.13 |
|  | Variance | <0.01 | 0.37 | <0.01 | 0.14 |
|  | Standard deviation | 0.09 | 0.61 | 0.08 | 0.38 |
| PMVS-P  (×10^6^) | Mean | 0.20 | 0.33 | 0.33 | 0.53 |
|  | Variance | 0.03 | 0.02 | <0.01 | 0.11 |
|  | Standard deviation | 0.16 | 0.13 | 0.08 | 0.33 |

**Table S1.** Mean, variance and standard deviation of fluorescence intensity of tumor recurrence after surgical resection in PBS, SAL, PMVS and PMVS-P groups at different time points. All values are accurate to two decimal places.
